# Research on cultural and creative design method of 2022 World Cup lamps based on AHP-FCE

**DOI:** 10.1371/journal.pone.0286682

**Published:** 2023-11-21

**Authors:** Tao Wang, HongZhu Chen, Basyarah Hamat, YanXiao Zhao

**Affiliations:** 1 Razak Faculty of Technology and Informatics, Universiti Teknologi Malaysia, Kuala Lumpur, Malaysia; 2 Anyang Institute of Technology Anyang, Henan, China; University of Belgrade Faculty of Organisational Sciences: Univerzitet u Beogradu Fakultet organizacionih nauka, SERBIA

## Abstract

**Aims:**

Through an in-depth study of Qatari culture, to explore the application of the essence of the unique national culture to World Cup creative design, and to provide new ideas and reference design framework and ideas for the integration of culture and World Cup creative design.

**Method:**

Carry out design practice with Qatari culture, and discuss in detail the specific strategy of integrating national culture into the cultural and creative design of the World Cup. First, conduct user interviews based on grounded theory to collect and evaluate demand indicators and establish a hierarchical model, and then use the Analytic Hierarchy Process (AHP) to analyze user needs, construct design elements for the World Cup cultural and creative design, and calculate the weight of each design element to determine the design Ordering among elements. Finally, the optimal scheme is selected by scoring the scheme through fuzzy comprehensive evaluation method (FCE), so as to determine the final design scheme of the product.

**Conclusion:**

The article explores the specific practice method of integrating culture and design, and provides a solution reference for how to integrate excellent national culture into the World Cup cultural and creative design, which not only improves the cultural and spiritual connotation of the product, but also effectively realises the heritage and innovation of culture.

## Introduction

The World Cup is one of the most influential sporting events in the world, with data showing that around five billion people participated in the 2022 FIFA World Cup in Qatar, with the final audience reaching nearly 1.5 billion. The World Cup, which relies on different countries, combines the history of the times, national cultural confidence, culture and art, and has a high research value. Its cultural and artistic value is mainly presented through cultural and creative products, architectural art and other methods, which not only reflect the connotation of the World Cup but also carry the essence of local culture, and have a certain value for the cultural dissemination and historical and cultural heritage of the World Cup. Shen D [1] etc. integrated the traditional culture of guqin into modern design, artistically processed the connotation of guqin culture according to social needs and application environment, and constructed the conceptual design of interactive guqin based on the three-level theory.;Bogucka E P [2] and others construct cultural maps based on cartographic narrative technology. The streets and buildings of the city have a unique historical culture of a city. By constructing a map with social and cultural characteristics, citizens’ historical awareness is improved.; Zhang X [3] et al. explored how to integrate cultural elements of architecture into cultural and creative product design based on perceptual engineering theory, optimizing the product design development process and promoting sustainable design;Dai Y [4] started with regional culture and landscape architecture design, explored the relationship between the two, and studied how to integrate regional culture into landscape architecture design, so as to provide theoretical reference for landscape architecture combined with regional culture design. The above-mentioned literature provides solutions to the development of regional cultural and creative design, but none of them has conducted sufficient user research, resulting in unclear design positioning and lacking certain theoretical guidance. Most studies are mainly analyzed from perceptual experience, lacking certain theoretical guidance. There is also a certain gap in deeply integrating local culture into cultural and creative design. The phenomenon of homogenization of design products is serious. The single addition of cultural shapes and symbols to products leads to superficial content and lack of modern design methods for regional cultural elements. Analysis, deconstruction and reorganization cannot reflect the added value of cultural and creative products [5], and generate emotional and cultural resonance with users. Therefore, this paper uses a combination of rooting theory, AHP and FCE methods to study how to apply national culture to World Cup cultural design. In the user research stage, most scholars establish evaluation indexes through questionnaires or expert interviews, while such methods are highly subjective and do not have certain theoretical support, thus ultimately causing large deviations to the evaluation results. Faced with the inadequacy of existing research methods, the rooting theory is understood. Rooting theory is a more scientific and reasonable qualitative research method, and its advantage lies in the distillation and generalisation of the original data, and then finding the essential problems of the research object. Many scholars have applied rooting theory, including Li Lin [6], who used it to analyse farmers’ willingness to sell their produce through new media and propose solutions and strategies, and Goodall K T [7], who used it to study how older people who “age in place” learn about information and digital technology. In addition, the use of semi-structured interviews in Root Theory is free-flowing and open-ended, and is not influenced by existing perspectives. Using AHP and FCE methods in the comprehensive evaluation process, Many existing scholars used AHP and FCE method individually or jointly for product design such as Çağlar Karamaşa [8] et al. used neutral AHP method to weight the best trainer indicators for FTO and solved the selection problem of trainers; Bakır M [9] et al. applied fuzzy AHP method for preference ranking of e-service quality criteria in the first phase of the study and accurately service quality assessment; Badi I [10] et al. combined AHP method with rough concept and applied to preference ranking of Libyan public railway industry, which improved the efficiency of selecting the best supplier in Libya; Alosta A [11] et al. used AHP to solve the complex planning problem of emergency medical aid stations in Libya; Zhou Z [12] et al. used AHP to conduct a study to compare and derive the influence sensitivity priorities of factors and optimized the application of human care factors in the design;S. Raja [13] et al. used AHP to solve the decision making problem in AMM selection; Sifan Guo [14] et al. used fuzzy comprehensive evaluation method to construct a fuzzy comprehensive evaluation model for community museum experience, which confirmed the practical value of the method in evaluating the quality of community museums; Xueyi Yu [15] et al. used fuzzy comprehensive evaluation to evaluate land reclamation, which provided ideas for the subsequent accurate evaluation of the comprehensive benefits of land reclamation; Yang X [16] used AHP and fuzzy comprehensive evaluation method to quantitatively calculate the fine-grained requirements, so as to obtain the influence of each requirement in design practice, lay the foundation for the theory of layered emotional design, and propose a more scientific and objective method. Research methods to alleviate the lack of emotional considerations in smart product design.;Zhang Y [17] et al. combined fuzzy comprehensive evaluation and analytic hierarchy process to determine index weights, and used fuzzy comprehensive evaluation method to perform matrix evaluation. Finally, according to the evaluation results, constructive suggestions were put forward for large-scale seawater desalination projects to promote the development of seawater desalination industry. AHP can be used to analyse the user requirements and weight the key requirements to indicate the design direction of the product, but the subjectivity factor is large. FCE can reduce the subjectivity of hierarchical analysis by integrating multiple evaluation indexes, and can evaluate the solution and select the optimal solution for further design improvement. Therefore, the author uses grounded theory to optimize the subjective deviation of the indicator determination process, then uses AHP to sort the indicators to determine the key needs of users, and finally uses FCE to optimize the program, in order to propose a decision that combines qualitative and quantitative methods Methods to improve the objectivity and scientificity of design and evaluation. This article applies Qatari culture to the World Cup cultural creation, combines cultural elements with product structure, and finally generates a culturally unique World Cup cultural creation. The purpose is to explore how to effectively and reasonably inject the unique essence of national culture into the World Cup cultural and creative design. , to provide new ideas for the World Cup cultural and creative design, and to provide new channels for the dissemination of national culture. This article provides a rational design method and evaluation process for the integration of culture into cultural and creative design, which is more concrete and feasible than the design of a perceptual experience method, and greatly reduces the gap between the real product and the real product on the ground, providing an excellent carrier for cultural heritage. The first part of this paper reviews and analyses existing research identifies problems and gaps in it, and then proposes more optimal solutions. It also reviews the application of these methods in different fields and studies and provides a literature review of them. Section two provides a detailed description of the methods used. Section three first obtains factor indicators through rooted theory analysis, performs hierarchical model construction, and then applies hierarchical analysis to obtain indicator weight rankings. Section four carries out a scheme design based on the ranking of indicator weights obtained in the previous section. Section five uses the fuzzy comprehensive evaluation method for scheme preference. Section six provides a discussion and section seven provides concluding remarks.

## Materials and methods

### Theoretical overview

#### Grounded theory

Grounded Theory was proposed by sociologists GLASER and STRAUSS [18] in 1967. Its main content is to establish a theory based on empirical data, summarize and gradually refine the data obtained from semi-structured interviews, and then rise to a systematic Theory, so as to abstract the core concept that can reflect the essence of things [19]. Grounded theory attaches great importance to data collection of empirical facts, and requires coding of all qualitative research data. Analysis and results must be based on data, requiring three-level coding of data, and emphasis on comparison and correlation between data [20]. Its workflow is problem selection, data collection, data analysis, theory establishment, theory saturation testing, and conclusion formation. The open, spindle, and selective coding are the three key steps in forming a theory. Grounded theory suggests that sampling and data collection should be continued until the domain is “theoretical saturated” with data. When the new data can neither generate new insights nor exceed the scope of the encoded data, it means that “theory and saturation” has been reached [21], otherwise, it is necessary to continue to collect data for analysis and verification.

#### Analytic hierarchy process

Analytic Hierarchy Process (AHP) is a systematic and hierarchical evaluation method proposed by American operations research expert Saaty in the 1970s [22]. communication barriers between them [23]. Analytic Hierarchy Process is a scientific method that combines qualitative and quantitative methods by comparing multiple target programs, decomposing complex problems into quantifiable target objects, and then summarizing [24]. The process is divided into three main steps:

1. Construct the index hierarchical model. The hierarchical model of the decision-making problem has several levels. The target layer is at the top, the middle is the criterion layer, and the bottom is the index layer [25].
2. Determine the weight vector of the criterion, score the criterion of each level by the expert group, summarize the data using the Delphi method [26], and normalize the comparison matrix through equation (1); then, calculate through equation (2) Normalize the average value of each row of the comparison matrix to obtain a weight vector: the expert group scores the criteria of each level, summarizes the data using the Delphi method, and establishes a judgment matrix for comparing all The importance between two elements is calculated subjectively.
  $$\begin{array}{c} {\bar{a}}_{ij}=\frac{a_{ij}}{\sum_{k=1}^{n}a_{ij}},ij=1,2,\ldots n, \end{array}$$ (1)
  $$\begin{array}{c} W_{i}=\sum_{j=1}^{n}\frac{\bar{a_{ij}}}{n},i=1,2...n. \end{array}$$ (2)
3. Consistency check. In order to ensure the consistency of the evaluator’s thinking in the process and the compatibility of the judgment matrix, it is necessary to conduct a consistency test on the evaluation results. Compute the largest eigenvalue:
  $$\begin{array}{c} \lambda_{\text{max}}=\frac{1}{n}\sum_{i=1}^{n}\frac{{\left(Aw\right)}_{i}}{w_{i}} \end{array}$$ (3)

Among them, n represents the order of the judgment matrix, and (*Aω*)_*i*_represents the product of matrix A and matrix. Then calculate the consistency index CI:

$$\begin{array}{c} CI=\frac{\lambda_{\text{max}}-n}{n-1} \end{array}$$ (4)

Calculation of CR value(see Table 12 for CI values):

$$\begin{array}{c} CR=\frac{CI}{RI} \end{array}$$ (5)

when *CR* < 0.1, the consistency check is passed, otherwise it is not passed, the matrix needs to be rebuilt and the consistency check is performed again until it passes. The values of RI are shown in Table 1.

**Table 1:** RI values of matrix order 1–9.

1	2	3	4	5	6	7	8	9
0	0	0.58	0.90	1.12	1.24	1.32	1.41	1.45

#### Fuzzy comprehensive evaluation method

The concept of fuzzy set was proposed by American automatic control expert Chad in 1965 [27]. It is a fuzzy comprehensive evaluation process. Its main process is as follows:

1. Establish the factor set. The factor set is a collection of various indicators, which are divided into two levels of indicator sets. The first-level index level is *U* = {*U*_1_, *U*_2_, *U*_3_…}, and the second-level index set is *U*_1_ = {*U*_11_, *U*_12_, *U*_13_…}
2. Determine the weight set. According to the analytic hierarchy process, all the expert evaluation results are summarized and calculated to obtain the weight. Afterwards, using the data collected in the questionnaire to further determine the weight of the secondary indicators with the weighted average method.
3. Create an evaluation set The evaluation set V is: *V* = {*V*_1_, *V*_2_, *V*_3_, …}, the evaluation level is divided into several ranges, and a certain score is assigned to each range.
4. Construction of fuzzy evaluation matrix Let R be a total evaluation matrix composed of n programs, and each of these programs is a combination of m indicators, *R* = (*r*_*ij*_)_*mn*_, namely:
  $$\begin{array}{c} R=[\begin{array}{cccc} r_{11} & r_{12} & ... & r_{1n}\\ r_{21} & r_{22} & ... & r_{2n}\\ ... & ... & ... & ...\\ r_{m1} & r_{m2} & ... & r_{mn} \end{array}] \end{array}$$ (6)
  Among them, *R*_*i*_ = (*r*_*j*1_, *r*_*j*2_, …*r*_*jn*_), (1, 2, …*m*) is the single-factor fuzzy evaluation set of the index *U*_*i*_, and the fuzzy evaluation set is a fuzzy subset on the evaluation set V;*r*_*ij*_ is The degree of membership of the comments *V*_*j*_ = (*j* = 1, 2, 3, 4, 5, 6) of the index *U*_*i*_ is determined after the corresponding calculation. When the weight *A*_*i*_ = (*a*_*i*1_, *a*_*i*2_, …*a*_*is*_) of the sub-factor set *U*_*i*_ = (*u*_*i*1_, *u*_*i*2_, …*u*_*is*_) is obtained according to the collected results, the fuzzy evaluation matrix of a certain factor is *B*_*i*_ = *A*_*i*_ × *R*_*i*_ = (*b*_*i*1_, *b*_*i*2_, …*b*_*in*_), and all single-factor evaluation matrices are:
  $$\begin{array}{c} R=[\begin{array}{c} B_{1}\\ B_{2}\\ \vdots \\ B_{n} \end{array}]=[\begin{array}{cccc} b_{11} & b_{12} & \ldots & b_{n}\\ b_{21} & b_{22} & \ldots & b_{2n}\\ \vdots & \vdots & & \vdots \\ b_{m1} & b_{m2} & \ldots & b_{nm} \end{array}] \end{array}$$ (7)
5. Secondary comprehensive evaluation Using the operator *U*_*i*_ = {*u*_*i*1_, *u*_*i*2_, …*u*_*is*_}, get:*B* = *A* × *R* = (*b*_1_, *b*_2_, …*b*_*n*_). Then the final solution score is: *W* = *B* × *V*^*T*^.

#### AHP/FBS method integration

AHP can analyze user needs and perform weight ranking according to key needs [28], Starting from the essential understanding of decision-makers on evaluation problems, it has more qualitative analysis and judgment than general quantitative research methods, but in saayt quantity When selecting values in the table, decision-makers are often hesitant to compare what they have done, so this method is subject to the subjectivity of researchers. but this method is subject to the subjectivity of researchers, while FCE can optimize multiple solutions, which reduces the subjectivity of evaluation to a certain extent at the decision-making level. Calculation deals with fuzzy evaluation objects, and the decisions made are more scientific and practical, which reduces the subjectivity of evaluation to a certain extent at the decision-making level. The combination of the two methods can not only systematically consider the influencing factors of the evaluation object, but also reduce the influence of subjective assumptions on the evaluation decision-making process [29]. avoiding the subjective will of decision-makers It is so strong that the product cannot express its basic semantics, so that various evaluation indicators can be quantitatively displayed, in order to propose a comprehensive evaluation method that combines qualitative and quantitative, so as to improve the objectivity and scientificity in the design and evaluation process. Designers provide theoretical reference, provide a new path for integrating the essence of national culture into the cultural creation of the World Cup, and better promote the inheritance of national culture.

### World Cup creative design process framework

**Fig 1:**
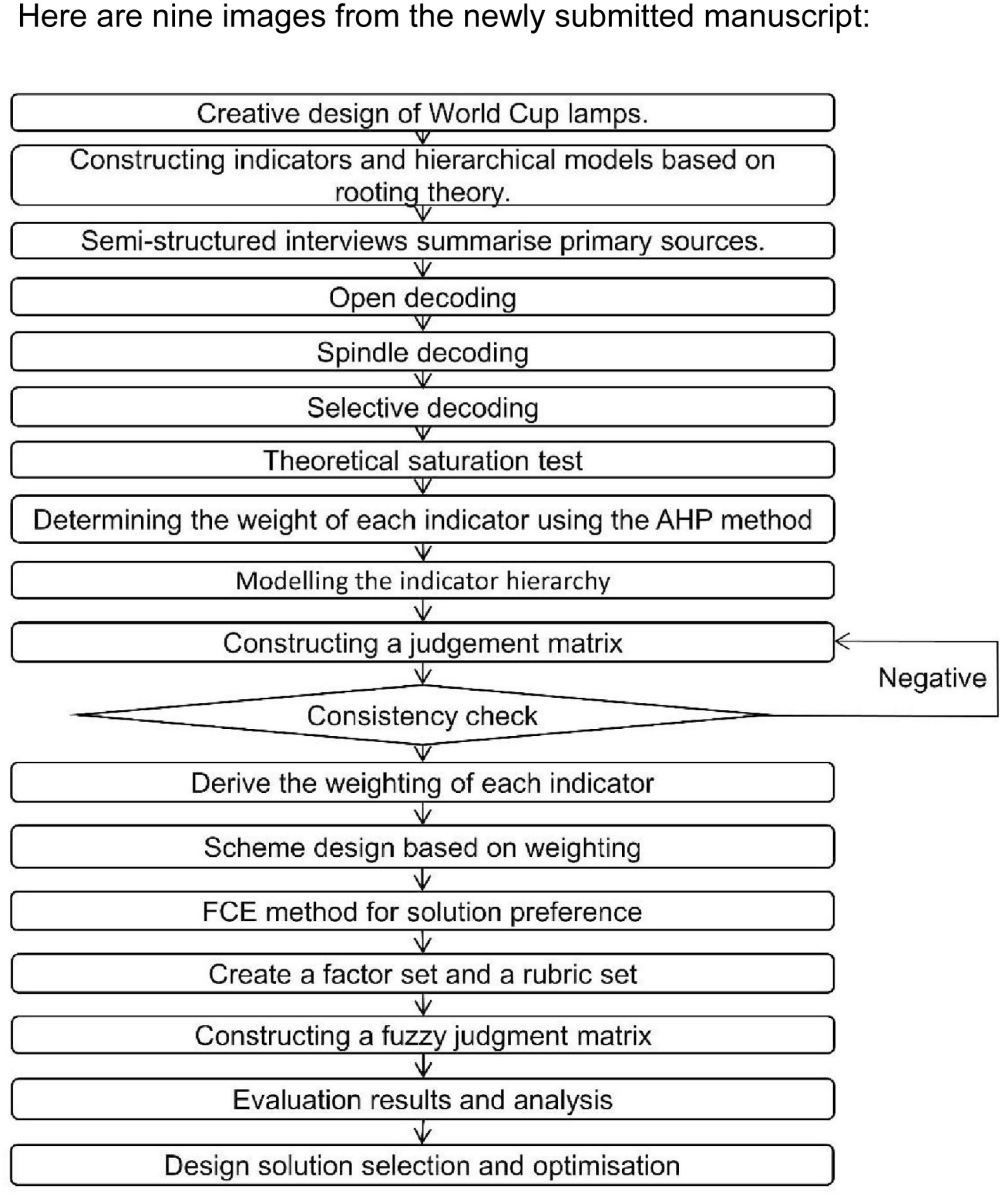
The comprehensive evaluation process of cultural and creative design for the World Cup.

This article is mainly an exploration of methods, and does not involve any relevant human research. Only human beings participated in the questionnaire survey, and the participants in the questionnaire survey were all volunteers. They were informed of the research purpose and interview process in advance and obtained the volunteers’ With written consent, all volunteers signed an informed consent form in advance, and all data were collected anonymously. In addition, there is no ethics committee in the author’s territory, so the study does not involve ethical violations. The overall process is divided into three stages, that is, the ranking of user demand indicators, program design and program screening and optimization, which are solved through the analytic hierarchy process and fuzzy comprehensive evaluation method. In the first stage, semi-structured interviews were conducted based on grounded theory, a hierarchical model was constructed, a judgment matrix was constructed, consistency checks were carried out, and the requirements were sorted by weight to obtain key requirements. In the second stage, the preliminary scheme design is carried out according to the key needs. In the third stage, the fuzzy comprehensive evaluation method is used to screen and optimize the scheme, so as to determine the final most perfect and optimal scheme. The overall process is shown in Fig 1.

## Results

### Construction of comprehensive evaluation of cultural and creative design of World Cup lamps and lanterns

#### Evaluation levels and indicators establishment

*Selection of the user sample*. Three product designers with five years’ experience or more, eight fans who had watched more than three World Cups (four of each sex, three in the age group 27–31, three in the age group 31–35 and two in the age group 35 or more; the degree of knowledge of cultural and creative products was classified as not knowing much and buying occasionally, more familiar and buying occasionally and familiar and buying often) and two historical and cultural In-depth interviews were conducted with the researchers, each lasting 25 minutes.

*Primary data collection*. In-depth interviews were conducted with the interviewees, based on a semi-structured interview form, during which the interviewer should not induce the interviewees but should guide them to answer the questions. The interview was audio-recorded after obtaining the consent of the interviewee to ensure that the information collected was accurate and that no information was missed. The interview questions are shown in Table 2.

**Table 2:** Semi-structured interview questions.

No	Questions
1	Do you often buy creative products?
2	What are your thoughts on incorporating cultural features into creative design?
3	Do you think they can convey a cultural message to a certain extent?
4	Do you have any demands on the shape and material of the products?
5	Do you expect interaction with the products?
6	Do you think there are any improvements to the function of the lamps?

*Open-ended decoding*. The recordings were transformed into text,using Nvivo12 to help decode the analysis, and in order to avoid the subjectivity of the interviewer, Open coding is the process of encoding, labeling, interpretation and induction of disordered original data, aiming to discover new insights from the original data phenomenon [30], so as to generate initial concepts. the original information was conceptualised, and the 12 categories were finally derived by summarising the original information several times and removing repetitive and unnecessary invalid information. The specific process is shown in Table 3.

**Table 3:** Open decoding process.

Raw material representative statements	Initial concept	Category
The keys are too loud for night use	Push button silent touch	Silent Touch
Not very comfortable to the touch, poor user experience	Lightweight, smooth and comfortable materials	Surface finish
Prefer the more fun kind	Enhances emotional experiences such as interaction	Fun and interactive
Structure is not very strong, easy to break	Structured and stable	Structural stability
Does not incorporate local cultural characteristics	Designed with local cultural characteristics	Geographical culture
Wish there was different brightness for different environments	Multiple brightness options	Brightness adjustment
The look is very homogeneous and not distinctive	Artistic shape	Unique shape
Too many function options, not easy to distinguish	Simple and straightforward operation steps	Easy to operate
No special features, just ordinary cultural creations	Incorporates elements that reflect cultural connotations	Use of cultural symbols
Some of the material breaks down after use, not good quality	Sturdy and durable materials	Durability of materials
The colours are fancy and unattractive	Simple and sensible use of colour	Colour matching
The light is too harsh and harmful to the eyes	Soft, unobtrusive lighting	Soft lighting

*Principal axis decoding*. This method is based on the analysis and collation of the original data to derive the respective categories, and then the main categories are derived according to the logical connections between the categories. The primary data obtained during the open decoding process is summarised and collated to produce four main categories, namely safety, culture, aesthetics and functionality. The detailed process is shown in Table 4.

**Table 4:** Spindle-based decoding process.

Main categories	Category	Inside
Security	Structural stability	Stable and solid structure of the luminaire, not easy to break
	Soft light	Soft light without glare
	Material Durability	Reasonable materials enhance the experience of use
Cultural	Regional culture	Culturally appropriate
	Use of cultural symbols	Clever use of cultural symbols to enhance culture
	Interesting interactivity	Appropriate interactivity for emotional connection
Aesthetics	Colour matching	Unique colour palette to match local character
	Unique styling	Unique shapes to highlight cultural characteristics
	Surface treatment	Appropriate finishes to enhance comfort
Functionality	Brightness adjustment	Different levels of brightness to suit different environments
	Silent touch control	Silent touch buttons
	Easy to operate	Easy to understand operating procedures

*Selective decoding*. The task of selective coding is to extract the most important core categories. By sorting out the main categories and their logical relations, further generalization and category integration are adopted. This method is a deep analysis of the categories obtained in the main axis decoding, looking for logical relationships between the categories and summarising their context and connotations. The specific process is shown in Table 5.

**Table 5:** Selective decoding process.

Typical path relationships	Nature of relationship	Connotations
Safety → user satisfaction → design solutions	Intermediary relationship	Safety and reliability affect user satisfaction and therefore design solutions
Cultural → user satisfaction → design solutions	Intermediary relationship	Culture affects user satisfaction and therefore design
Aesthetics → user satisfaction → design solutions	Intermediary relationship	Aesthetics influence user satisfaction and therefore design solutions
Functionality → user satisfaction → design solutions	Intermediary relationship	Functionality affects user satisfaction and therefore design solutions

*Theoretical saturation test*. The theoretical saturation test is used to determine the confidence and validity of the theoretical study [31], and the model is proved to be theoretically saturated by using the three original data set aside for coding analysis again until no new concepts or categories appear.

**Fig 2:**
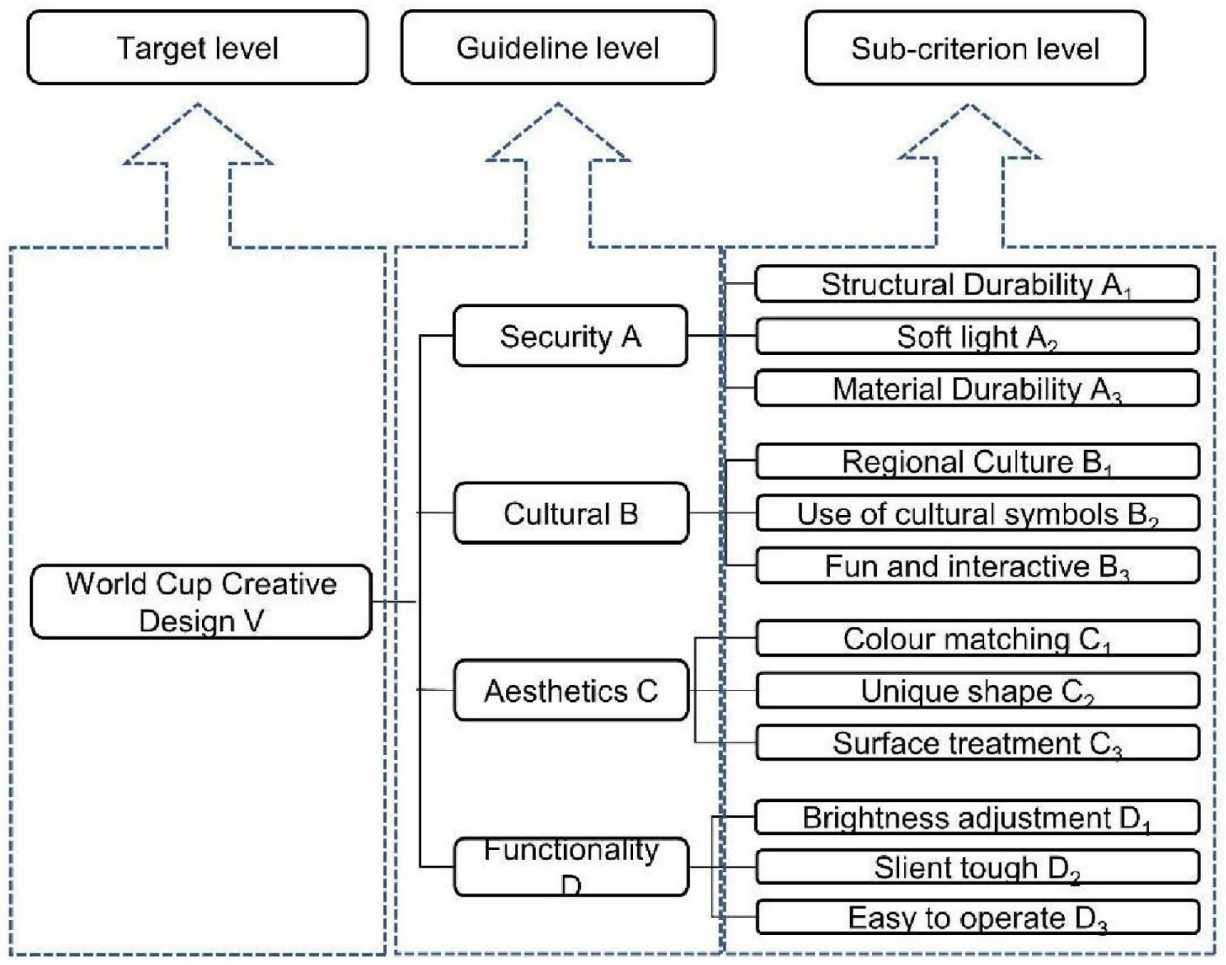
World Cup luminaire design hierarchy model.

*Indicator hierarchy model construction*. Through the design research of World Cup cultural creation, 4 main categories and 12 sub-categories were used as indicators for evaluation, and finally the indicator hierarchy model of World Cup luminaire cultural creation was established, as shown in Fig 2.

#### Constructing a judgement matrix

To obtain the weight of each requirement, a matrix was constructed to compare the requirements of the same tier with each other, and integers from 1 to 9 were used [32](shown in Table 6) to rate the importance of each requirement against each other. The user samples selected above were used to score each tier, and eventually the results were analysed several times to bring the results into agreement and hence the requirement weights.

**Table 6:** Scale of judgment matrix importance indicators.

Scale	Level of importance	implication
1	Equally important	Indicator i and indicator j are equally important
3	Slightly important	Indicator i is marginally more important than indicator j
5	Significantly important	Indicator i is significantly more important than indicator j
7	Very important	Indicator i is very important compared to indicator j
9	Absolutely important	Indicator i is more important than indicator j
2,4,6,8	Median value	Take the middle part
Countdown	Inverse comparison	If the importance of indicator “i” to indicator “j” is “n”, the opposite is “1/n”.

#### Results

The matrix analysis of the various levels of the World Cup luminaire cultural and creative design gives the weight values of each indicator. According to the analysis in Tables 7 to 12, the highest weight in the criterion level is cultural (0.4696), followed by safety (0.2584), functionality (0.1553) and finally aesthetics (0.1167). The sub-criteria with the highest weighting were regional cultural (0.2576), softness of light (0.1522), use of cultural symbols (0.1131), ease of operation (0.1006), fun and interactive (0.0990) and structural stability (0.0651). Through the above data, it can be found that when cultural and creative products are integrated with cultural elements, they have a special spirituality and cultural nature of cultural products. With the improvement of people’s living standards, cognitive ability and increasing demand for cultural products, people pay more and more attention to The cultural value of cultural and creative products, that is, its ideological value, artistic value, etc., is also the added value of the product [33], which can bring people a deeper level of spiritual pleasure. Therefore, in the cultural and creative design of World Cup lamps and lanterns, the cultural use and safety guarantee of lamps and lanterns should be considered. During the design process, Qatar’s regional culture should be deeply excavated and analyzed, typical cultural elements should be extracted for redesign, and cultural elements should be organically combined with the cultural and creative design of the World Cup. In terms of safety, attention should be paid to the structural stability of the product and the durability of the material.

**Table 7:** Judgement matrix and weight values for the target layer.

V	A	B	C	D	Weights w
A	1	1/3	3	2	0.2584
B	3	1	4	2	0.4696
C	1/3	1/4	1	1	0.1167
D	1/2	1/2	1	1	0.1553

**Table 8:** Judgement matrix and weights for safety criteria.

A	*A*_1_	*A*_2_	*A*_3_	Weights w
*A*_1_	1	1/3	2	0.2519
*A*_2_	3	1	3	0.5889
*A*_3_	1/2	1/3	1	0.1593

**Table 9:** Judgement matrix and weights for the culturalness criterion.

B	*B*_1_	*B*_2_	*B*_3_	Weights w
*B*_1_	1	2	3	0.5485
*B*_2_	1/2	1	1	0.2409
*B*_3_	1/3	1	1	0.2106

**Table 10:** Aesthetic criteria matrix and weights.

C	*C*_1_	*C*_2_	*C*_3_	Weights w
*C*_1_	1	1/3	2	0.2884
*C*_2_	3	1	1	0.4484
*C*_3_	1/2	1	1	0.2632

**Table 11:** Functional criteria matrix and weights.

D	*D*_1_	*D*_2_	*D*_3_	Weights w
*D*_1_	1	2	1/3	0.2299
*D*_2_	1/2	1	1/5	0.1222
*D*_3_	3	5	1	0.6480

**Table 12:** Judgement matrix and weights for each indicator.

Guideline level	Weights	Sub-criterion layer	Weights	Combined weights	Sort by
A	0.2584	*A*_1_	0.2519	0.0651	6
		*A*_2_	0.5889	0.1522	2
		*A*_3_	0.1593	0.0412	9
B	0.4696	*B*_1_	0.5485	0.2576	1
		*B*_2_	0.2409	0.1131	3
		*B*_3_	0.2106	0.0990	5
C	0.1167	*C*_1_	0.2884	0.0337	11
		*C*_2_	0.4484	0.0523	7
		*C*_3_	0.2632	0.0307	10
D	0.1553	*D*_1_	0.2299	0.0357	8
		*D*_2_	0.1222	0.0190	12
		*D*_3_	0.6480	0.1006	4

#### Consistency test

As can be seen from Table 13, the consistency indicators for each tier are less than 0.1, indicating that the consistency test is passed and the weighting values are reasonable.

**Table 13:** Consistency test results.

Consistency indicators	V	A	B	C	D
λ_max_	4.160	3.054	3.018	3.054	3.004
CI	0.053	0.027	0.009	0.027	0.002
RI	0.890	0.520	0.520	0.520	0.520
CR	0.060	0.052	0.018	0.052	0.004

λ_max_ is maximum characteristic root; CI is the consistency index;RI is a random consistency index CR is the consistency ratio.

### Design practice

#### Qatari culture

Qatar is located on the Qatari Peninsula on the southwestern coast of the Persian Gulf, surrounded by sea on three sides and bordering the Persian Gulf, at the crossroads of sea and desert. Its capital, Doha, is the largest city and the economic, transport, and cultural center of the country, and is one of the most famous ports in the Persian Gulf. Culturally, Qatar is a country of tradition and modernity, with a maritime, religious, tourist, and technological culture that reflects the diversity of the local culture. In particular, the maritime tradition of Qatar’s cultural heritage is of great importance to the country and is reflected in the country’s new coat of arms. These diverse cultures together form a country with a distinctive cultural identity.

#### Refinement of cultural elements

**Fig 3:**
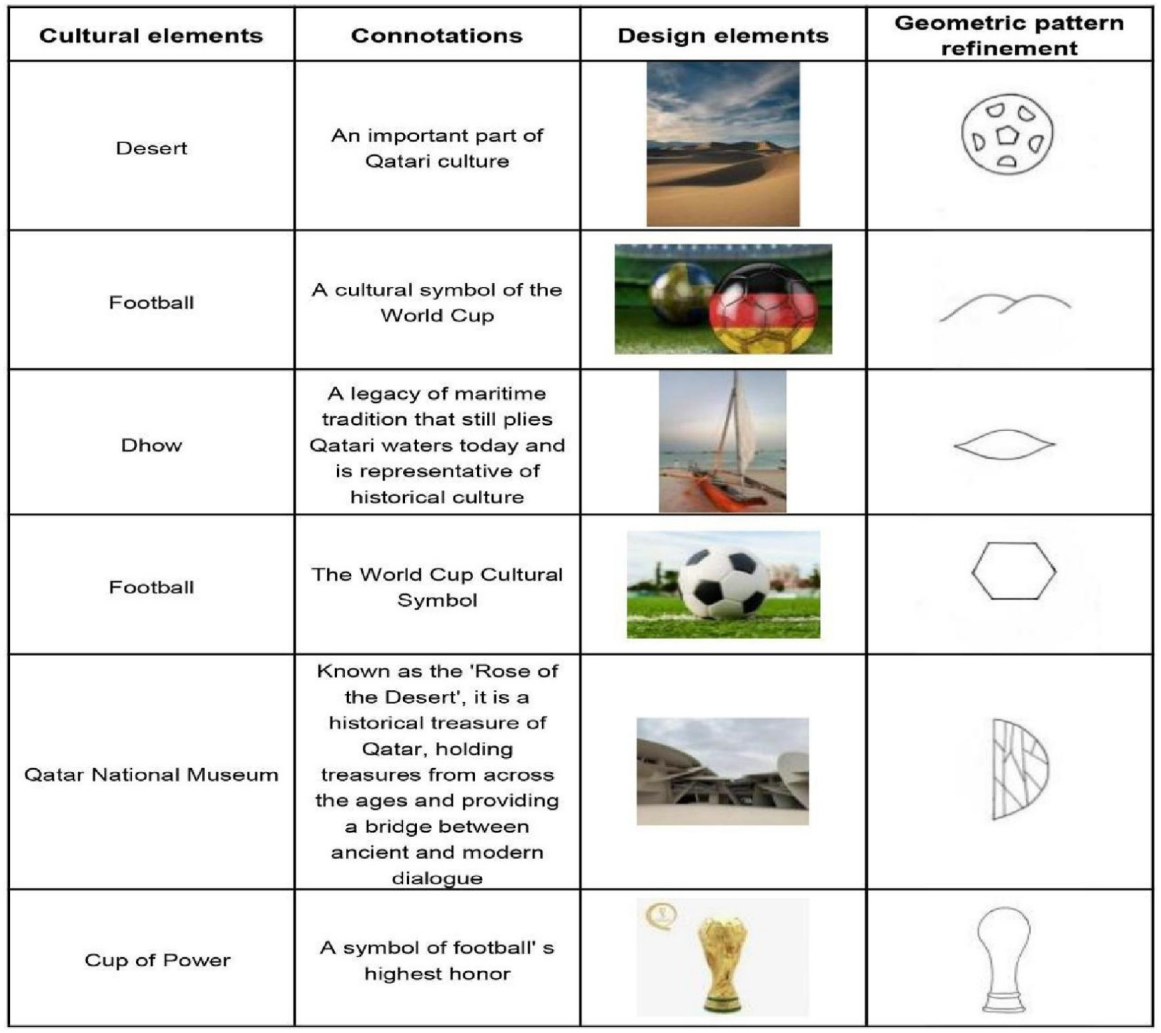
Extraction of cultural elements.

Based on an in-depth analysis of the Qatari culture, the desert, the dhow, and the Qatar National Museum were selected as cultural elements. Based on the theory of morphological perception in the visual arts, the most prominent features are the first to be captured when people recognize form, and the human eye tends to see any observed object as the simplest shape that known conditions allow to be achieved [34]. Therefore, the three cultural features are used as representatives to further abstract them into simple patterns or geometric shapes, thus stimulating associations and a sense of cultural charm in the subtleties. The distillation of the cultural elements is shown in Fig 3.

#### Programme design

Through the research on the local culture of Qatar, the design elements are derived from the cultural connotation, the representative cultural elements are extracted, and the hidden features are combined with the use and interaction of the product by using metaphor, symbol, exaggeration and other techniques, and integrated as added value Among the design elements of cultural and creative products. In the creative design of cultural products, it is necessary to comprehensively consider the final impact of various elements on the product, such as product appearance aesthetics and product connotation. The product appearance is mainly reflected in the product shape, surface texture, etc.; It mainly includes two dimensions of semantic layer and connotation layer. The semantic layer reflects its essential meaning and spiritual outlook, which is the specific emotion integrated into the creation. The connotative layer refers to the cultural connotation and implication behind the product, which is deeper information such as history, and reflects the spiritual core of the times. And various complex images are abstracted into recognizable simple geometric patterns, which not only retains the characteristics of characteristic design elements, but also has a certain degree of innovation, making the cultural elements reasonable And properly apply it to cultural and creative products.

**Fig 4:**
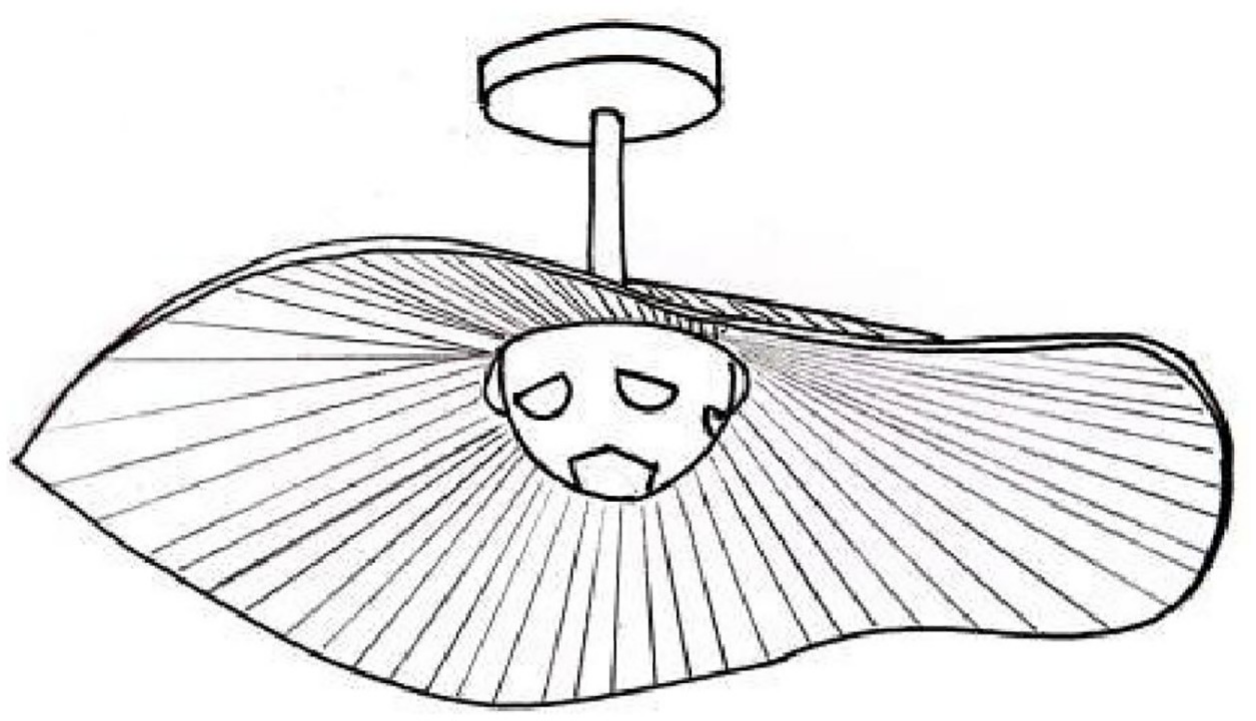
Preliminary programme.

**Fig 5:**
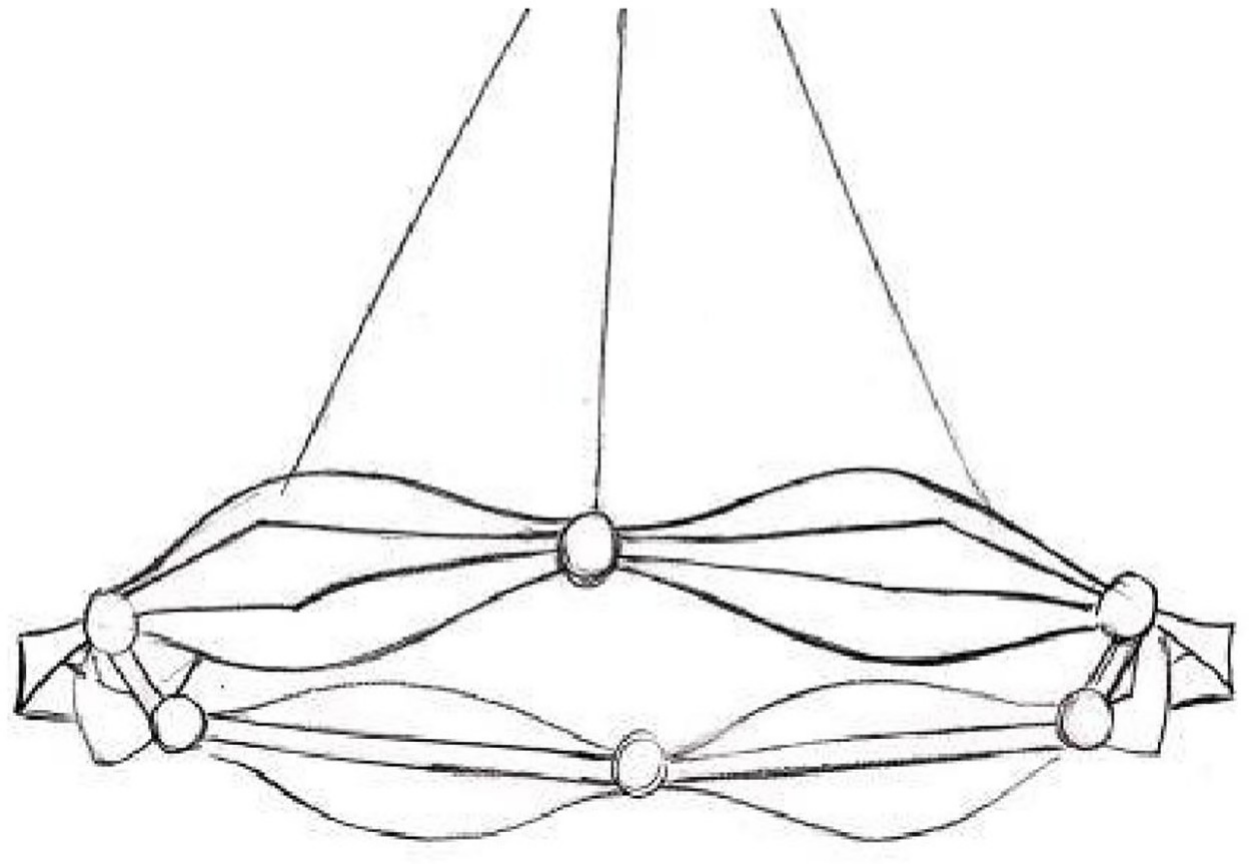
Extraction of cultural elements.

**Fig 6:**
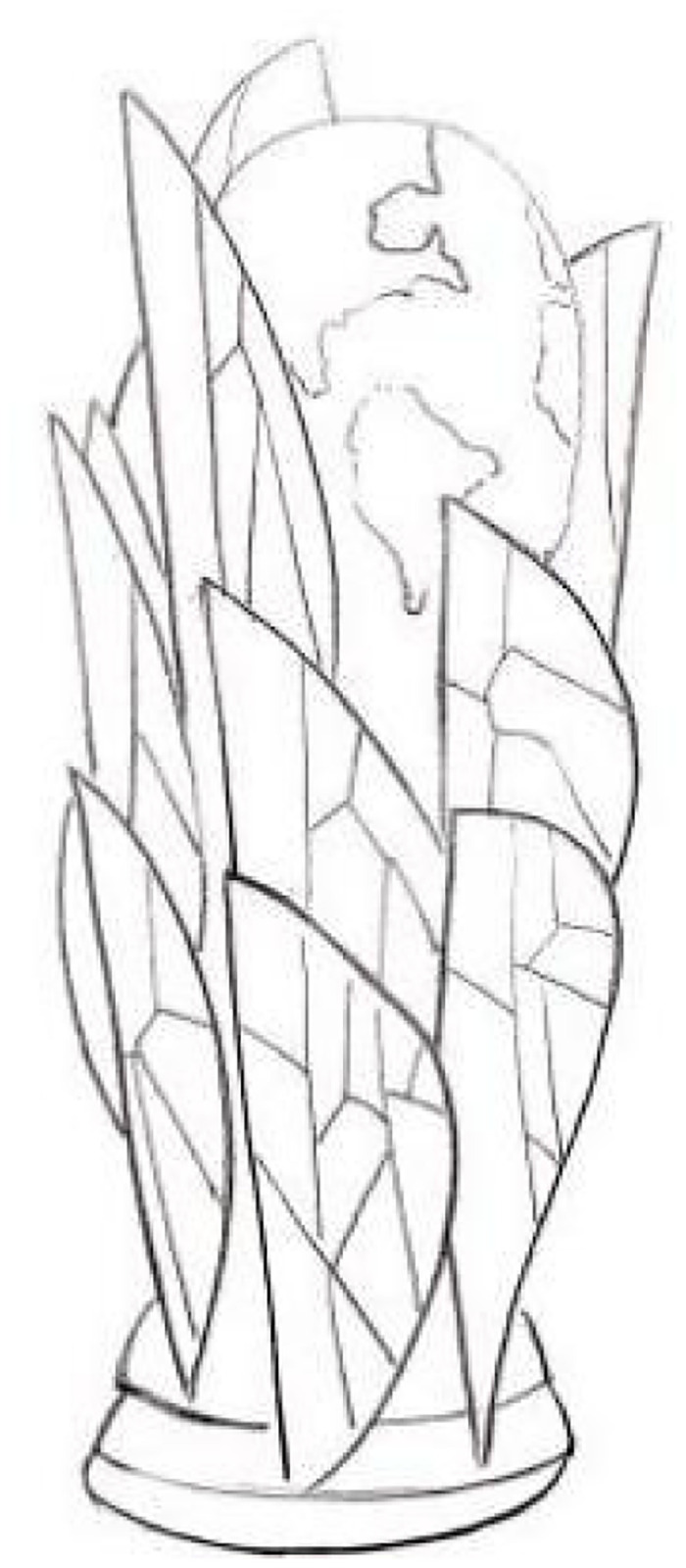
Extraction of cultural elements.

1. Option 1
  The scheme is a geometric abstraction of the World Cup football, taking its shape as the light source theme, while the external shape is a reference to the desert culture of Qatar, inspired by the undulating lines of the desert. The shape does not prohibit the rolling sand dunes and the World Cup culture.
2. Option 2
  The scheme takes the form of a wooden dhow sail and combines it with the hexagonal shape of a World Cup football as the main design element. The outer hexagonal lines of the sail are wavy, resembling the waves of the sea, and the wooden material used for this part gives the visual impression of a sailing ship moving on the sea. The redesigned wooden hexagon and the sail together express the long history of Qatari maritime culture.
3. Option 3
  The scheme consists of two parts, the exterior is the shape of the Qatar National Museum, inspired by the desert rose, taking design elements from the National Museum and abstracting them into the simplest of geometric patterns, thus creating a culturally integrated design. Inside is the Hercules Cup, a symbol of the World Cup. The integration of Qatar’s desert culture into the World Cup cultural and creative design effectively achieves cultural heritage and innovation, and also provides a new channel for the dissemination of national culture. Fig 3 shows preliminary sketches of the three options. the final plan is shown in Figs 4–6.

### Fuzzy integrated evaluation

#### Fuzzy comprehensive evaluation method

FCE can effectively solve the complex problems in World Cup luminaire cultural and creative design that cannot be quantified due to environmental factors and subjective factors of designers, and the model is simple and easy to understand [35] The specific steps are as follows.

1. Invite a sample of users in the index evaluation to participate in the evaluation. The criterion index is used *a* = {*a*_1_, *a*_2_, *a*_3_, *a*_4_}, which refers to safety, culture, aesthetics and functionality respectively. The sub-criteria are *a*_*i*_ = *a*_*ij*_(*i*, *j* = 1, 2, 3)
2. Establish evaluation levels and scoring criteria. Determine the evaluation set *V* = {*v*_1_, *v*_2_, *v*_3_, *v*_4_}=very satisfactory, satisfactory, average, unsatisfactory and specify a very satisfactory score of 90–100; satisfactory score of 75–90; average score of 60–75; and unsatisfactory less than 60.
3. According to Tables 6–10, we can obtain the weight values of indicators at each level, the criterion level *W*_*A*_ = (0.2548, 0.4696, 0.1167, 0.1553);*W*_1_ = (0.2519, 0.5889, 0.1593);*W*_2_ = (0.5485, 0.2409, 0.2106);*W*_3_ = (0.2884, 0.4484, 0.2632);*W*_4_ = (0.2299, 0.1222, 0.6480)

For the sub-criteria layer for each scheme fuzzy comprehensive evaluation matrix to be constructed. *D*_1_ represents the evaluation matrix of the safety criterion layer for Scheme 1; *D*_2_ represents the evaluation matrix of the cultural sub-criterion layer for Scheme 1; *D*_3_ represents the evaluation matrix of the aesthetic sub-criterion layer for Scheme 1; *D*_4_ represents the evaluation matrix of the functional sub-criterion layer for Scheme 1. matrix. The results are as follows.

$$\begin{array}{c} D_{1}=[\begin{array}{cccc} 0.4 & 0.3 & 0.3 & 0\\ 0.4 & 0.2 & 0.4 & 0\\ 0.3 & 0.3 & 0.4 & 0 \end{array}]\\ D_{2}=[\begin{array}{cccc} 0.5 & 0.2 & 0.3 & 0\\ 0.2 & 0.4 & 0.4 & 0\\ 0.2 & 0.3 & 0.5 & 0 \end{array}]\\ D_{3}=[\begin{array}{cccc} 0.2 & 0.4 & 0.4 & 0\\ 0.2 & 0.5 & 0.3 & 0\\ 0.3 & 0.3 & 0.4 & 0 \end{array}]\\ D_{4}=[\begin{array}{cccc} 0.2 & 0.4 & 0.4 & 0\\ 0.2 & 0.3 & 0.5 & 0\\ 0.6 & 0.3 & 0.1 & 0 \end{array}] \end{array}$$

From the single-indicator fuzzy composite evaluation matrix, the evaluation weight vector for Option 1 at the criterion level is calculated as follows:

$$\begin{array}{c} P_{1}=Wa_{1}\times D_{1}=(\begin{array}{cccc} 0.384 & 0.241 & 0.375 & 0.000 \end{array})\\ P_{2}=Wa_{2}\times D_{2}=(\begin{array}{cccc} 0.365 & 0.269 & 0.366 & 0.000 \end{array})\\ P_{3}=Wa_{3}\times D_{3}=(\begin{array}{cccc} 0.226 & 0.419 & 0.355 & 0.000 \end{array})\\ P_{4}=Wa_{4}\times D_{4}=(\begin{array}{cccc} 0.459 & 0.323 & 0.218 & 0.000 \end{array}) \end{array}$$

The combined weights are then

$$P=[\begin{array}{c} P_{1}\\ P_{2}\\ P_{3}\\ P_{4} \end{array}]=[\begin{array}{cccc} 0.384 & 0.241 & 0.375 & 0\\ 0.365 & 0.269 & 0.366 & 0\\ 0.226 & 0.419 & 0.355 & 0\\ 0.459 & 0.323 & 0.218 & 0 \end{array}]$$

The overall evaluation weight of the World Cup luminaire design is:

*W* = *W*_*A*_ × *P* = (0.368, 0.288, 0.344, 0) From the above calculation process, it can be seen that the percentage score for Option 1 is 75.36, similarly Option 2 is 81.81 and Option 3 is 78.44. As shown in Figs 4–6, Option 2 is therefore the best solution. Option 2 was further refined and designed. (The calculation data of the remaining two schemes can be seen in the attachment).

#### Scheme refinement design

**Fig 7:**
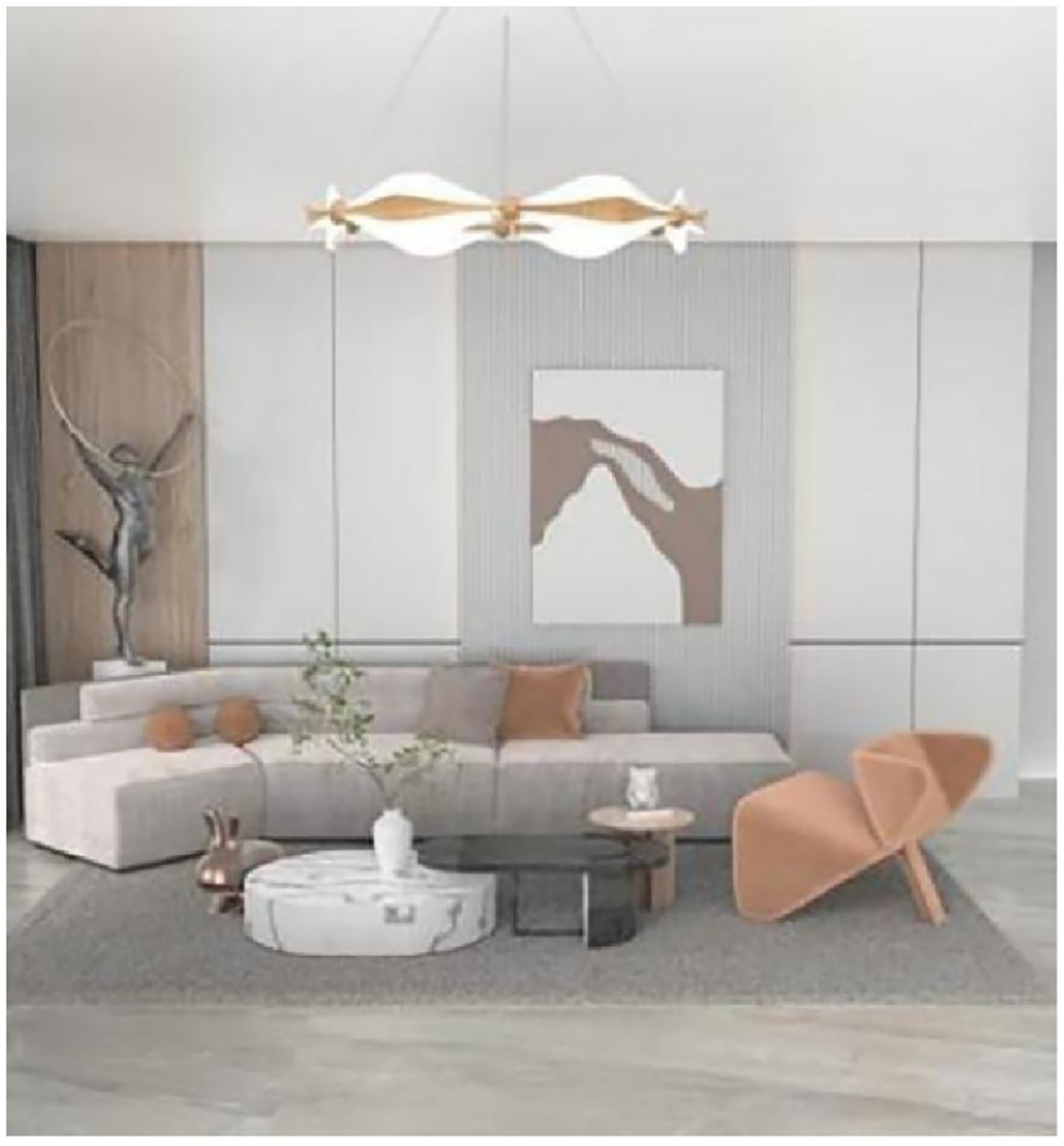
Creative design of World Cup lamps.

**Fig 8:**
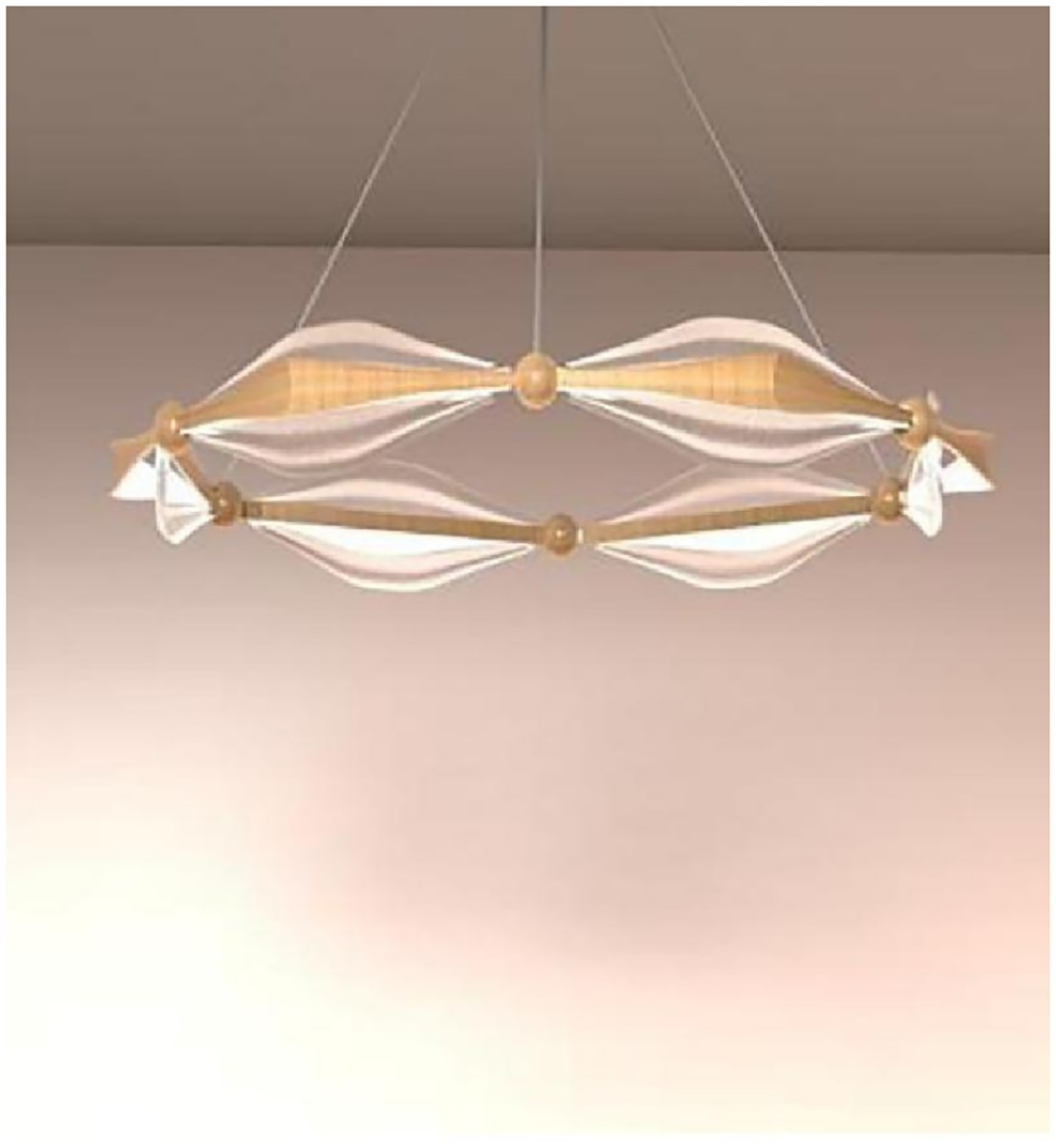
Extraction of cultural elements.

**Fig 9:**
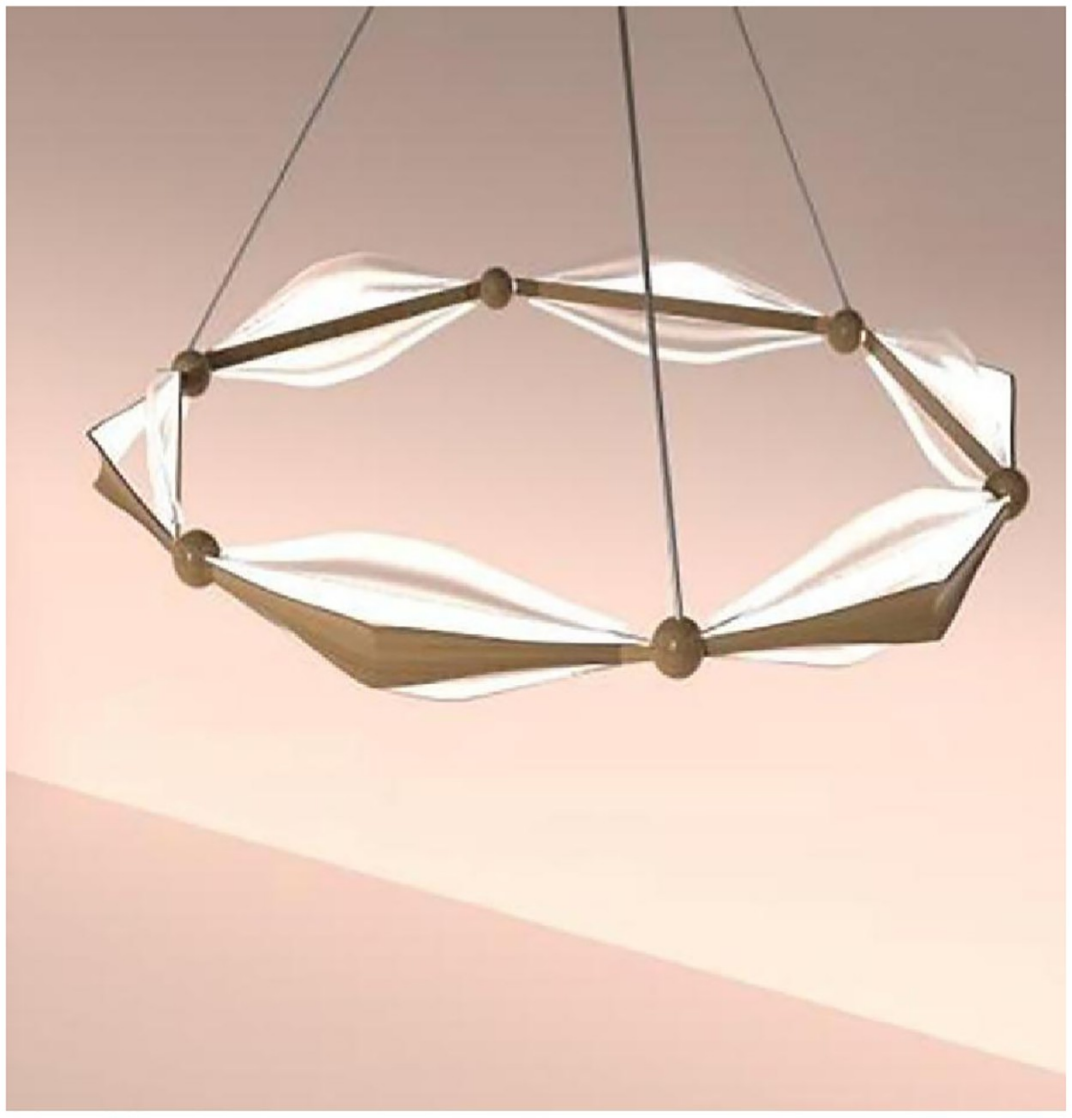
Extraction of cultural elements.

The design of the scheme takes into account the inseparable relationship between Qatar and the sea, and the fact that wooden dhows [36], a legacy of Qatari maritime culture and tradition, are still plying Qatari waters today, and therefore takes the sail form of a wooden dhow and combines it with the hexagonal shape of a World Cup football as the main design element. The outer hexagon has been stylistically modified with wavy lines, the surface of the sail is textured with the surface of the World Cup Janub stadium, together expressing the long and important history of seafaring, and the inner hexagon acts as a fixation and symbol of the World Cup culture at the same time. In terms of materials, both hexagonal frames are made of wood, echoing the traditional wooden dhow, while the sails are finished in Plexiglas. The overall shape is simple and fluid, rich in cultural connotation yet aesthetically pleasing, as shown in Figs 7–9.

## Discussion

The World Cup is the most influential international sporting event in the world, relying on different countries to hold it, and is a natural cultural carrier. Through an in-depth study of Qatari culture, the essence of its unique national culture is explored and typical cultural elements are extracted to subtly blend the national culture with the World Cup. Regional culture is unique and unrepeatable because it reflects the historical development of the nation, its customs and beliefs [37]. In-depth excavation of national cultural elements can not only awaken people’s sense of cultural identity, but also protect and inherit culture to a certain extent, and make national culture vital through contemporary design methods. Based on the grounded theory, this paper screens the indicators and constructs the hierarchical model, uses the analytic hierarchy process to sort the key needs of users, and finally uses the fuzzy comprehensive evaluation method to optimize the scheme. The article integrates the three methods for design innovation and program evaluation, which is more scientific, rigorous and convincing than the design results purely using the perceptual experience method, and is more objective than using only the AHP, using grounded theory and fuzzy synthesis The evaluation method effectively reduces the subjectivity of decision makers and makes them more rigorous and reasonable. Integrating national culture into the design of the World Cup, extracting typical elements of Qatari culture through modern design, the national culture can be reasonably and skillfully integrated into the World Cup cultural creation, providing new ideas and new methods for the design of the World Cup cultural creation. At the same time, it promotes and provides channels for the dissemination of national culture. The paper still has many limitations. Firstly, the focus of this paper is on how to apply the national culture to the design of the World Cup cultural creation, and the calculation method is relatively single. Therefore, it is possible to combine multiple calculation methods in the future to make the evaluation results more reasonable and accurate. Secondly, the interviewees were not carefully categorised, and factors such as educational level and the degree of development of the region in which they lived could affect their understanding of a product, thus producing different results. Therefore, in the follow-up experiments, the interviewees can be carefully divided, and more targeted questions can be used for interviews. The next stage of this research is to compare a variety of different design and decision-making methods, select the easiest and most objective method, further improve and optimize the plan, and provide new ideas for the integration of cultural elements into the World Cup cultural and creative design and cultural promotion.

## Conclusion

In this paper, we use full user research, hierarchical analysis and fuzzy comprehensive evaluation method to construct a hierarchical model from four aspects: safety, culture, aesthetics and functionality, calculate the weight of each indicator and rank them according to their importance, design a scheme according to the top-ranked indicators, then score the three schemes with the help of fuzzy comprehensive evaluation method to filter out the best scheme, and finally optimize and improve the design. The article provides a theoretical reference for the integration of national cultural creativity into the cultural and creative design and development of the World Cup, which reduces the gap between product design and reality to a certain extent, therefore, local culture can be effectively disseminated through the medium of cultural and creative products,to provide new channels for cultural transmission. In the follow-up investigation, we should continue to dig deeper into other major factors affecting users, expand the number of survey participants, combine the development trend of cultural and creative products, and continuously innovate World Cup cultural and creative products.

## Supporting information

S1 File (DOC)

S1 Dataset (DOCX)

S1 Appendix (DOCX)

S1 Table (DOCX)
