# Correction: Research on cultural and creative design method of 2022 World Cup lamps based on AHP-FCE

**DOI:** 10.1371/journal.pone.0316861

**Published:** 2024-12-30

**Authors:** Tao Wang, HongZhu Chen, Basyarah Hamat, YanXiao Zhao

There are errors in the author affiliations. The correct affiliations are as follows:

Tao Wang^1,2^, HongZhu Chen^2^, Basyarah Hamat^1^, YanXiao Zhao^1^

**1** Razak Faculty of Technology and Informatics, Universiti Teknologi Malaysia, Kuala Lumpur, Malaysia, **2** Anyang Institute of Technology, Anyang, Henan, China.
