# Supplementary material for: Research on cultural and creative design method of 2022 World Cup lamps based on AHP-FCE
Source: PLoS One. 2023 Nov 21;18(11):e0286682. doi: 10.1371/journal.pone.0286682 (PMC10662743; doi:10.1371/journal.pone.0286682)
Supplement: S1 File — (DOC) [file pone.0286682.s001.doc]

**Content of the interview**

The research covers three categories of people: World Cup fans, product designers and historical and cultural researchers. The following is the research process.

**World Cup fan1**

**Occupation: Freelancer**

**Familiar with cultural and creative products and buy them regularly.**

I often buy some creative products, such as blind boxes, handicrafts, small ornaments and so on. I have a special collection of anime and movie figures, which are very memorable. I think the material should be safe and comfortable to touch, but some products break down after use and are not of good quality. If it's just a simple decoration, it doesn't have much value or character. I think that a cultural creation is only a carrier, but it can convey a cultural message, and a cultural creation that incorporates cultural characteristics may make people more interested in buying it, as well as enriching the value of the product itself. I think I would prefer something that is more fun and multifunctional. I had a lamp before, but it was a push button switch, so if I wanted to use it at night, I might have to adjust it or something, and the sound would be very loud, which was quite disturbing to others.

**World Cup fan 2**

**Occupation: Education practitioner**

**Familiar with cultural and creative products and a frequent buyer.**

I have bought a Forbidden City lipstick before, and I think it is a good combination of Forbidden City culture and cultural creation. Now I want to have all six. The quality of some things varies. I bought a ceramic mug a fortnight ago, but the paint fell off within a few days, and some things are easily broken because of their unstable structure. I would choose to buy creative products if they look good, have some value and are of good quality. It would make sense to design something interactive and the experience would definitely be better. I would like to see the functions of the World Cup luminaire be simple and clear, as there are too many functions to distinguish. It would also be more artistic if the shape was unique.

**World Cup fan 3**

**Profession: Blogger**

**More familiar and occasional purchases.**

I occasionally buy some cultural products as gifts for people. Some things can only be used as decoration, but when they are bought, they are actually just sitting there collecting dust, not of any practical use, so I usually don't buy them. If you want to give it to a friend, you will consider whether it is interesting or not. Young people like interesting and novel things, and most of the things you see on the internet are similar in feeling, so every time you look at them, you get a little aesthetic fatigue. A combination of culture and design would definitely be more meaningful than a stand-alone design, and could convey a cultural message to a certain extent. I hope that the design can be more artistic, and that the structure should be strong, sometimes the parts fall off by courier, which may be a quality problem. The World Cup luminaires are basically similar in function, and it would be good to look better in terms of shape.

**World Cup fan 4**

**Occupation: Freelancer**

**Not familiar with cultural and creative products, occasional purchases.**

Buy occasionally, not specifically known about it. Applying culture to creative design is a new idea, and have seen some great creative designs that have a soul. It combines two style elements into one and they complement each other. So the combination of culture must be appropriate and in line with the aesthetics of today's youth. You want the product to be interactive and interesting. The shape should be aesthetically pleasing, the material is not special. The World Cup luminaire design can be functional with different options for light intensity and type.

**World Cup fan 5**

**Occupation: Education practitioner**

**I am familiar with football products and buy them occasionally.**

I don't go out of my way to buy cultural products, but occasionally I will search Taobao when I need them. Incorporating culture into creative design is a new selling point, just like education can't be taught rigidly anymore, the combination of diversity definitely makes people more willing to buy, because people are relatively unfamiliar with the cultural output, but it might be better if it is presented in an appropriate way. It is certainly expected that the products are interactive and can be played with when you are bored, which is also a way to relax. It is better to have different brightness options for the function of the light fixture and the light must be soft and not harsh.

**World Cup fan 6**

**Occupation: Freelancer**

**More familiar with football cultural and creative products and occasional purchaser.**

I occasionally buy a little bit of something like this for decoration, and I know a little bit about it. It's good to incorporate cultural features into the design of the creations, which is quite popular nowadays. Not only does it provide ornamental value but it also shows cultural knowledge, so you can kill two birds with one stone. The shape can be designed with reference to the local culture, and the material is as smooth and comfortable as it can be. For the interactive cultural creations will be more like. The World Cup lighting design should still show the spirit of the World Cup, after all, it is also the youth of our generation, it is best not to be too complicated in terms of function, too much will not be able to distinguish which is which, simple is good.

**World Cup fan7**

**Occupation: Company employee**

**Familiar with football culture products and a regular buyer.**

I often buy this kind of creative products, I buy all kinds of them, most often some jewellery. The combination of culture and design can enrich the value of the creations themselves, and many of the things I buy are combined with culture, some of them are very well made, they look good and have an ethnic flavour, but there are also some that just have a different design, which is actually the same as ordinary creations, without actually incorporating cultural characteristics. If you want to buy something to decorate, you want it to be interactive and interesting.

**World Cup fan8**

**Occupation: Education practitioner**

**Not familiar with football culture products, buy occasionally to try .**

I don't buy creative products very often and don't know much about them. Combining cultural features and creative design can spread cultural values and also innovate products. I would like to see an interaction between the product and us. The shape should not be too exaggerated and the material should be mild and not easily broken. The main function of the lamp is to adjust the brightness, and it would be better if the warm and cold tones of the light could be adjusted.

**Product Designer 1**

**Years of experience：8 years**

**Occupation: product designer**

Cultural and creative products are in fact the productisation of cultural concepts, allowing consumers to feel the presence of culture while buying the product. It is important to find out the differences between cultural products and other products in order to design unique and meaningful products. Other products focus more on the practicality, materials and structure of the product, while creative products generally combine practicality and culture, so culture is the added value. To put it plainly, products that are interesting, artistic and practical are more popular. The majority of people who buy cultural and creative products are young people, who are increasingly interested in the spiritual side of things, so the role of cultural and creative products is to meet the spiritual side of things. The most important thing is to get people to recognise the product from the heart. Nowadays, the homogenisation of creative products in the market is serious, and under the banner of cultural integration, they are actually no different from ordinary creative products, which are used to attract users, but do not really deliver culture. For example, the Henan Museum has designed an archaeological blind box with its own Luoyang shovel, which allows consumers to dig up cultural relics at home, providing new ideas for integrating culture into creative design. Doing so truly awakens historical and cultural confidence in people's hearts. Good creative design allows cultural relics to cease to be dusty history and enter our daily lives in a multifaceted way. This is the true meaning of creative design. Nowadays, creative products tend to have a single shape, without their own characteristics, and do not really combine local culture and creative products in a clever way. Products that are interactive are of course better, like the Henan Museum's archaeological blind box that allows consumers to experience the process of archaeology with a certain amount of fun. The point is still to allow consumers to feel the cultural charm in a subtle process. The design of the World Cup lamps must first of all be a clever integration of culture into the design of the lamps, functionally the light must not be too harsh, after all this is the most important demand of the consumer, and secondly it is best to have different lights to adapt to different scenarios of use.

**Product Designer 2**

**Years of experience：5 years**

**Profession: Product Designer**

Nowadays, it is no longer enough to have a good look for a creative product, which means that new elements need to be explored and combined with the product to bring it to life. This means that new elements need to be explored and combined with creative products. A good cultural product can be an effective cultural and content export. For incorporating culture into products, I believe that three characteristics must be fulfilled: aesthetics, functionality and connotation, one of which is indispensable. It is not advisable to design products without any characteristics, more homogeneous or without a clear design point, or to design them in a very strange way to attract consumers' attention. The key is to bring the product into everyday life, with a sense of history and modernity, artistry and entertainment, which is the way to develop our cultural and creative products, and to make culture truly 'come alive'. The interactivity of the products can enhance the emotional connection between culture and consumers, and many excellent cultural and creative designs resonate on a spiritual level. In terms of materials, I don't think there are any special requirements. We suggest that the design should be based on local craftsmanship and materials, but it must be of good quality, as many designs have some quality problems in the process of use, such as being easily broken or not very strong. Lamp design in function is some details of the processing, different brightness adjustment, can be touch control will have a better experience.

**Product Designer 3**

**Years of experience：6 years**

**Profession: Product Designer**

In order to do well, it is an inevitable trend to integrate culture into creative products, to return to traditional culture, to find innovation in tradition, but also to keep up with the times, based on modernity, the product should be in line with the habits of contemporary people, give the product a sense of truth, so that the design can impress consumers. In fact, many products are now only under the banner of cultural integration, but actually use the brand to attract users, the product itself is very perfunctory, no characteristics and poor experience. To make a really good cultural design, it is important to highlight the regional culture, follow the consumers' daily life and reduce the value of the brand's commercial interests. The product also needs to focus on its interactivity, to be able to communicate with people in order to be popular. Of course, the same effect can be given in the colour scheme. A reasonable colour scheme will bring people different psychological feelings. The shape needs to have a certain sense of art and beauty, so that people can see the desire to buy, the material needs to be analyzed on a case-by-case basis, but must be safe and environmentally friendly. The function of the lamps is firstly to set different brightness levels on the light source, which can be used in different environments, and secondly to reflect certain cultural characteristics, which need to be tapped into the local cultural characteristics, to extract the elements and apply them in the design to the lamp design.

**Historical and Cultural Researcher 1**

**Years of practice: 15 years**

**Occupation: Researcher at the Chinese Academy of Social Sciences**

This is a very good start. The fusion of culture and art means that we can document diverse cultures, literature and religions through artwork, and the integration of national essence into creative design also opens up new paths for the transmission of history, more in line with the mainstream thinking and aesthetics of today's youth, making it easier for people to accept and learn about cultural history. It is easier for people to accept and learn about culture and history. In order to truly spread culture, it is necessary to carefully study the connotations and meanings of culture and history, and extract its essence to design beautiful cultural and creative products. It is best to make the products interactive, so that history and culture can really come to life not only with a doll, but also with personal experience, dialogue with the ancients and a real sense of cultural charm. In terms of shape, a secondary design can be made with reference to historical buildings, household items and so on, in line with current trends. The lighting design should ideally have a wider choice of lighting atmospheres and need not be set in a complex way.

**Historical and Cultural Researcher 2**

**Years of practice: 10 years**

**Occupation: University professor**

Usually, I buy some cultural and creative products. In today's rapid development of the Internet, when people enjoy material abundance and the convenience of life, they gradually forget the importance of inheritance, and cultural creation provides such an opportunity. It is an opportunity to use the material as the carrier and the spirit as the connotation, to restore and preserve the traces of the past and to awaken the national spirit deep inside. They have their own unique understanding of the products, and it is important to learn to study the design from these perspectives in order to come up with a new path of their own. In terms of shape, it is important to have your own unique features, not the same, and to think about what angle to innovate from. The design function of the lamp can have intelligent voice control or touch screen, and the light should not be too harsh.
