# Supplementary material for: Research on cultural and creative design method of 2022 World Cup lamps based on AHP-FCE
Source: PLoS One. 2023 Nov 21;18(11):e0286682. doi: 10.1371/journal.pone.0286682 (PMC10662743; doi:10.1371/journal.pone.0286682)
Supplement: S1 Dataset — (DOCX) [file pone.0286682.s002.docx]

The following is the weight and final score of the second and third schemes in section 5.1 calculated by the fuzzy comprehensive evaluation method:

1. Scheme 2

Count the number of times users score each index of the sub-criteria layer, get the relevant membership degree of each index, and then construct the fuzzy matrix. R_1_ represents the evaluation matrix of the safety criterion layer on the second scheme; R_2_ represents the evaluation matrix of the cultural sub-criteria level on the second scheme; R_3_ represents the evaluation matrix of the aesthetic sub-criteria level on the second scheme; R_4_ represents the evaluation matrix of the functional sub-criteria layer for the second scheme. The result is as follows:


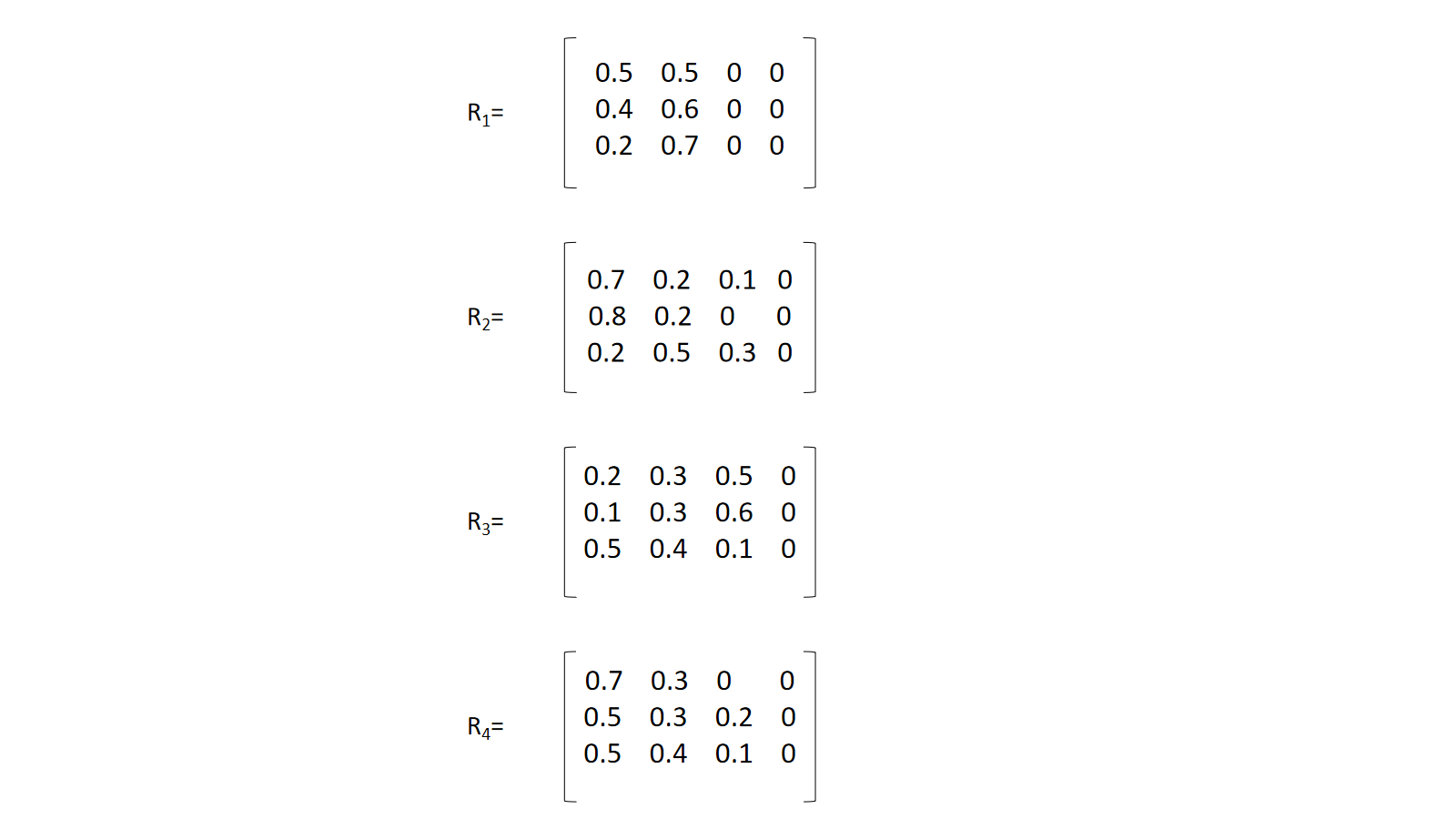


Calculated from the single-index fuzzy comprehensive evaluation matrix, and according to the multiplication-bounded operator, the evaluation weight vector of the criterion layer for scheme 1 is obtained as follows:

T_1_=W_b1_×R_1_=(0.397 0.587 0.016 0.000)

T_2_=W_b2_×R_2_=(0.672 0.245 0.083 0.000)

T_3_=W_b3_×R_2_=(0.325 0.350 0.325 0.000)

T_4_=W_b4_×R_4_=(0.582 0.333 0.085 0.000)

Then the comprehensive weight is:


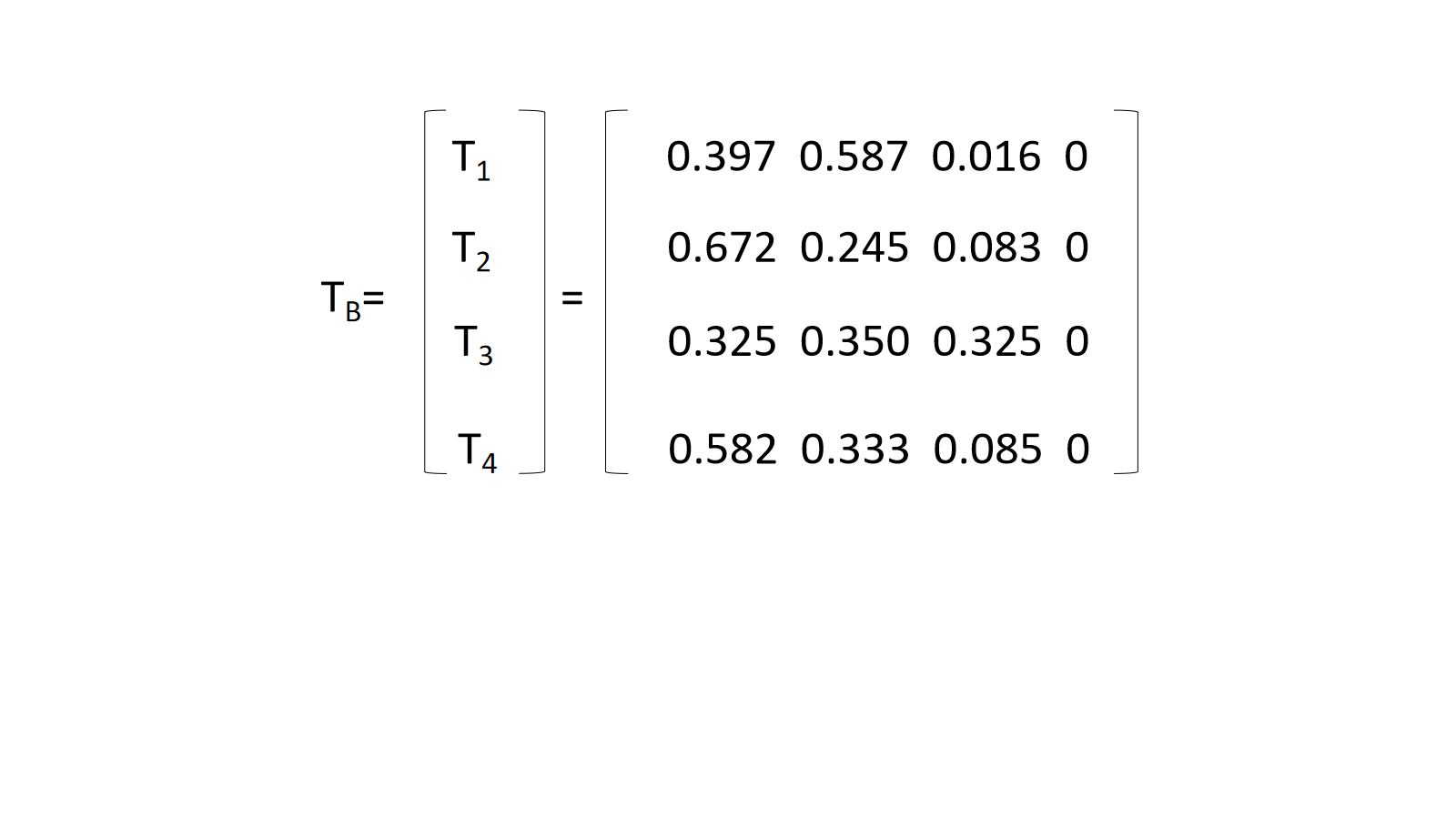


The comprehensive evaluation weight of cultural and creative design of World Cup lamps and lanterns is:

W=W_B_×R=（0.549 0.356 0.095 0）

From the above calculation process, the percentile scoring result of Scheme 2 can be obtained as 81.81.

2. Scheme 3

Count the number of times users score each index of the sub-criteria layer, get the relevant membership degree of each index, and then construct the fuzzy matrix. R_1_ represents the evaluation matrix of the safety criterion layer for the third scheme; R_2_ represents the evaluation matrix of the cultural sub-criteria level for the second scheme; R_3_ represents the evaluation matrix of the aesthetic sub-criteria level for the second scheme; R_4_ represents the evaluation matrix of the functional sub-criteria layer for the second scheme. The result is as follows:


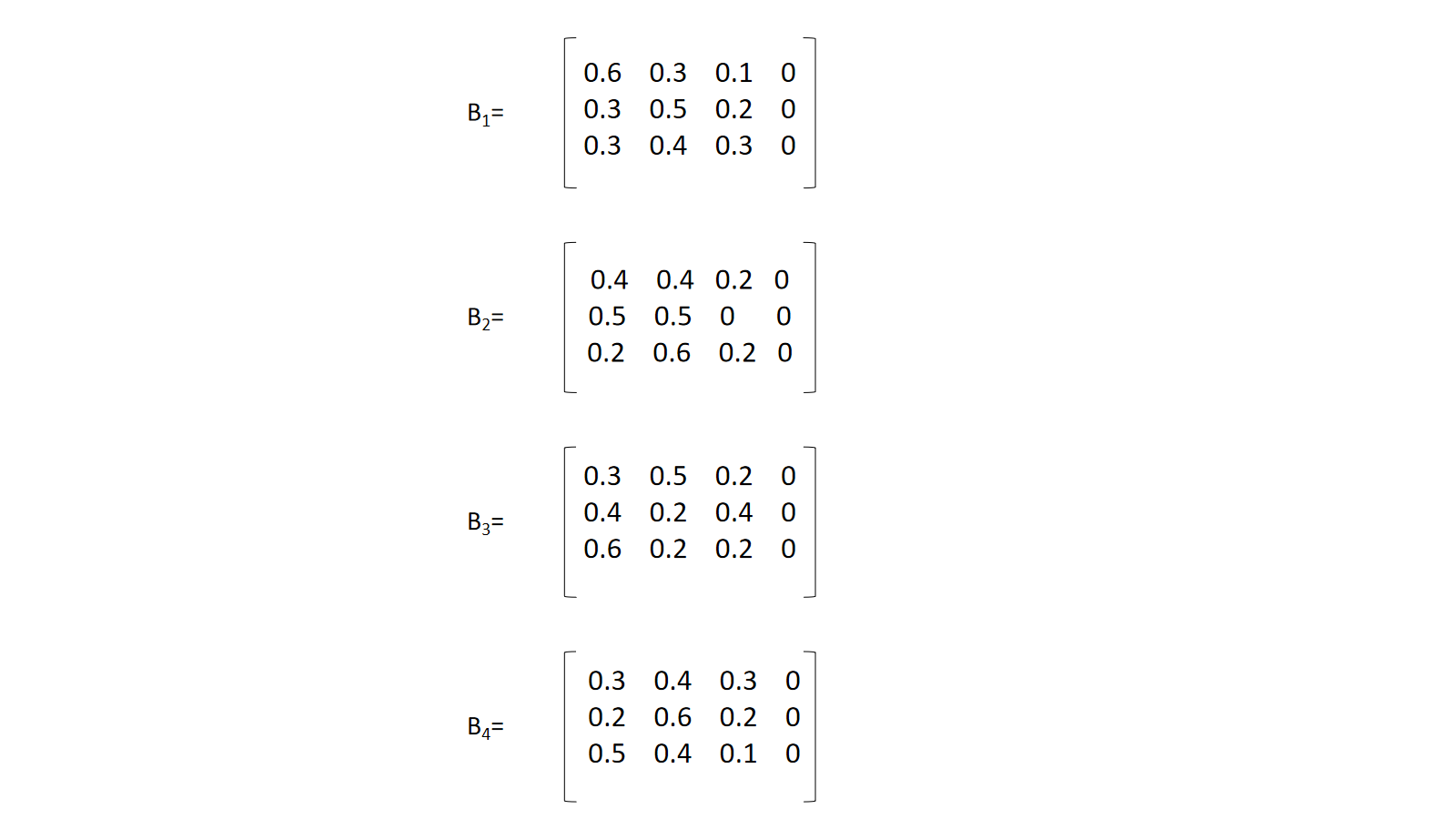


Calculated from the single-index fuzzy comprehensive evaluation matrix, and according to the multiplication-bounded operator, the evaluation weight vector of the criterion layer for scheme 1 is obtained as follows:

P_1_=W_c1_×B_1_=(0.446 0.426 0.128 0.000)

P_2_=W_c2_×B_2_=(0.458 0.467 0.075 0.000)

P_3_=W_c3_×B_3_=(0.403 0.289 0.308 0.000)

P_4_=W_c4_×B_4_=(0.306 0.508 0.186 0.000)

Then the comprehensive weight is:


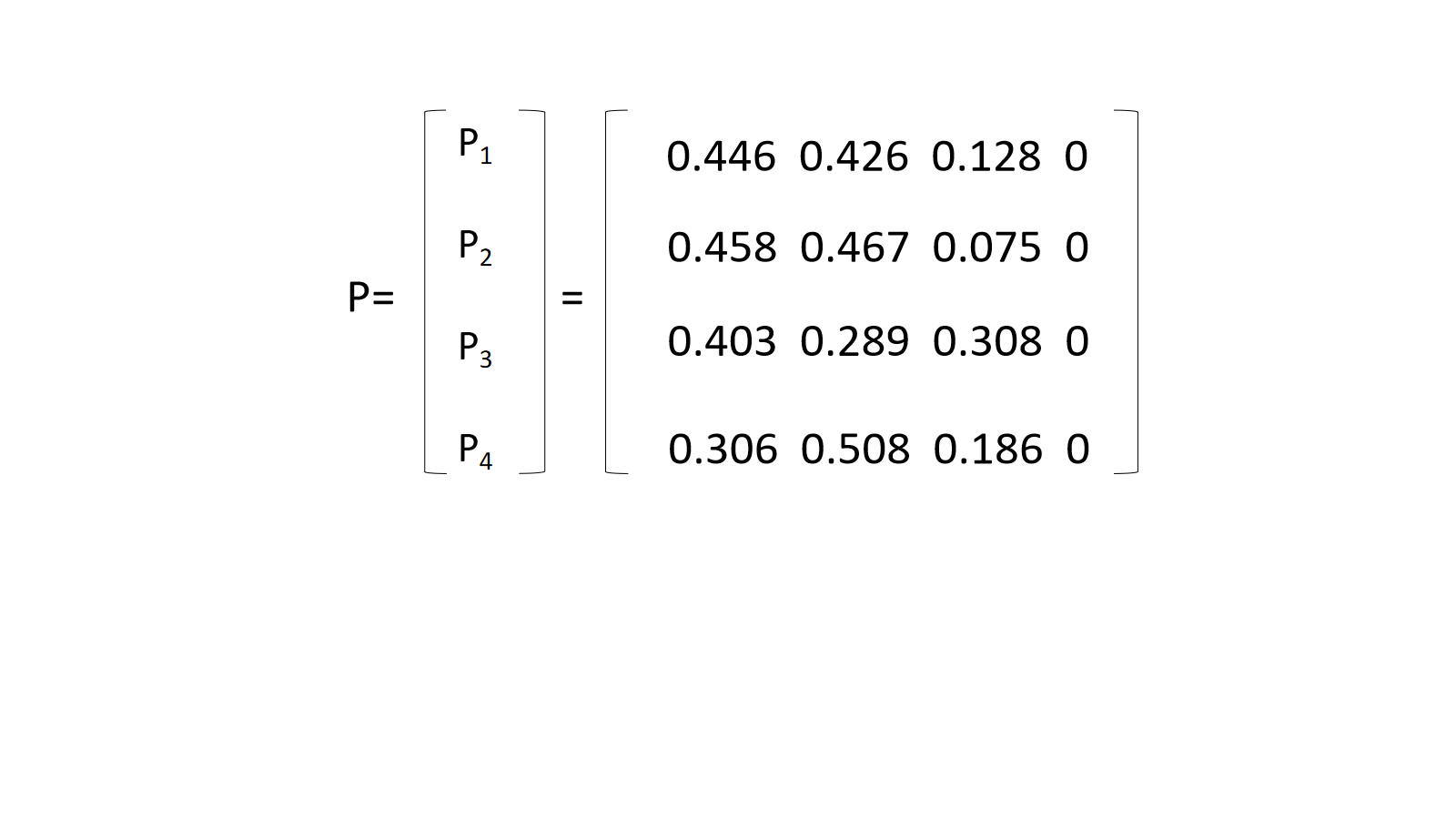


The comprehensive evaluation weight of cultural and creative design of World Cup lamps and lanterns is:

W=W_C_×P=（0.403 0.423 0.174 0）

From the above calculation process, the percentile scoring result of scheme three can be obtained as 78.44.
