# Supplementary figures and images for: Research on cultural and creative design method of 2022 World Cup lamps based on AHP-FCE
Source: PLoS One. 2023 Nov 21;18(11):e0286682. doi: 10.1371/journal.pone.0286682 (PMC10662743; doi:10.1371/journal.pone.0286682)

Below are the tables and figures from the manuscript:


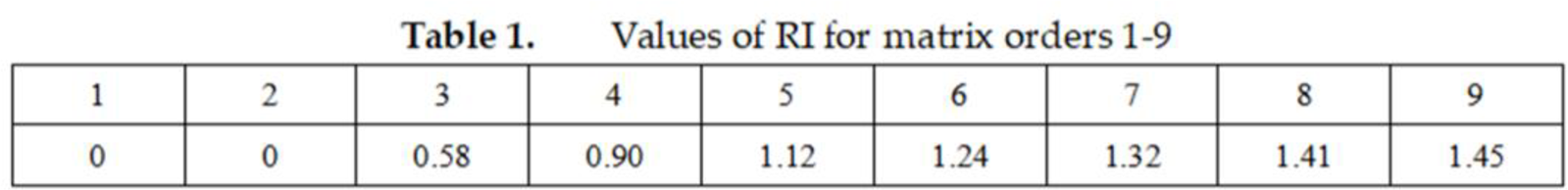


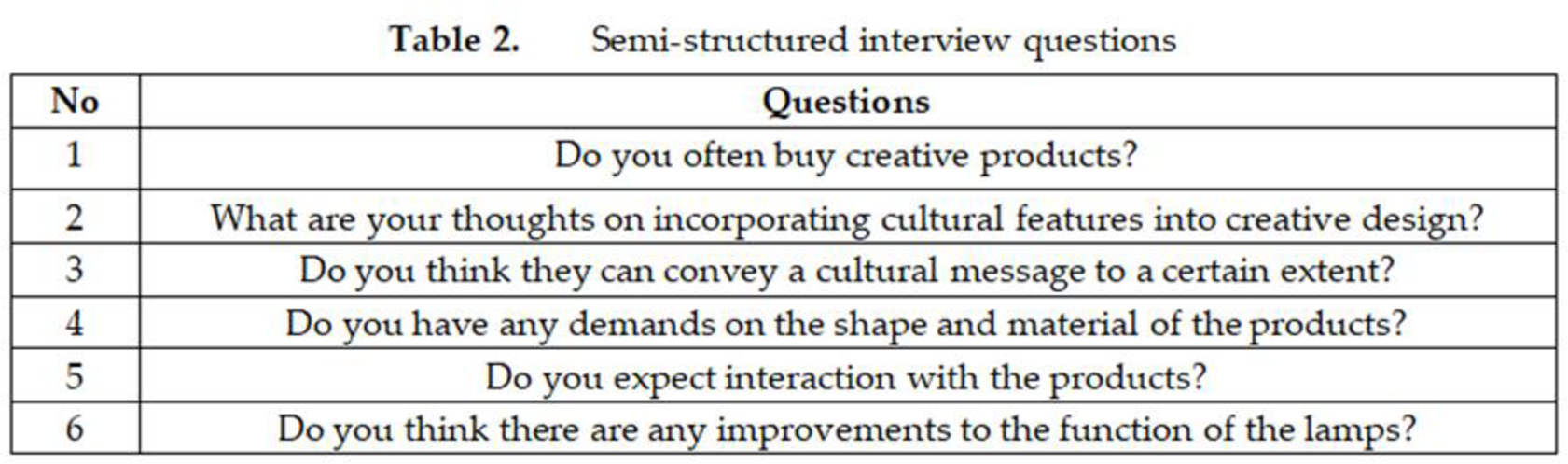


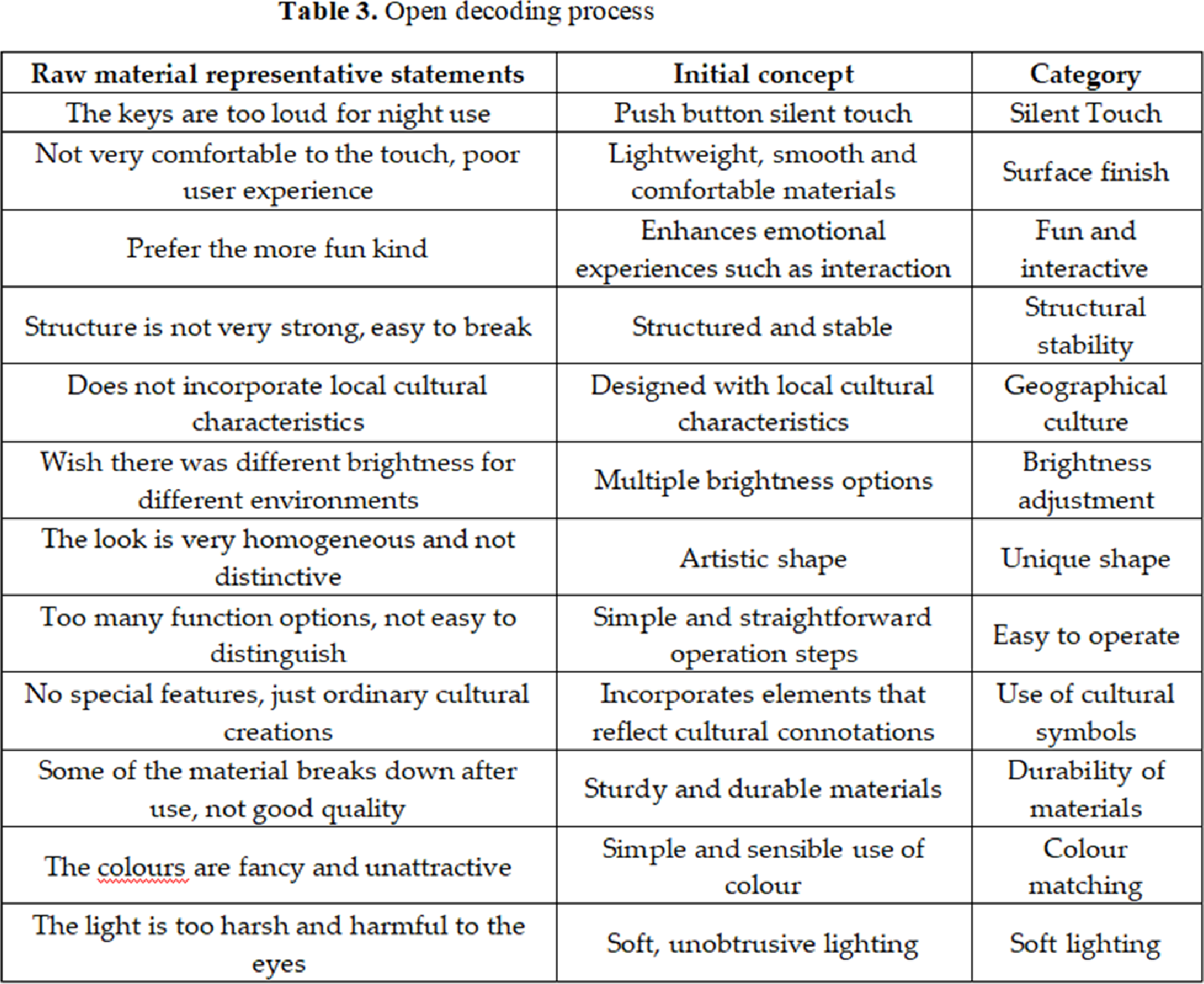


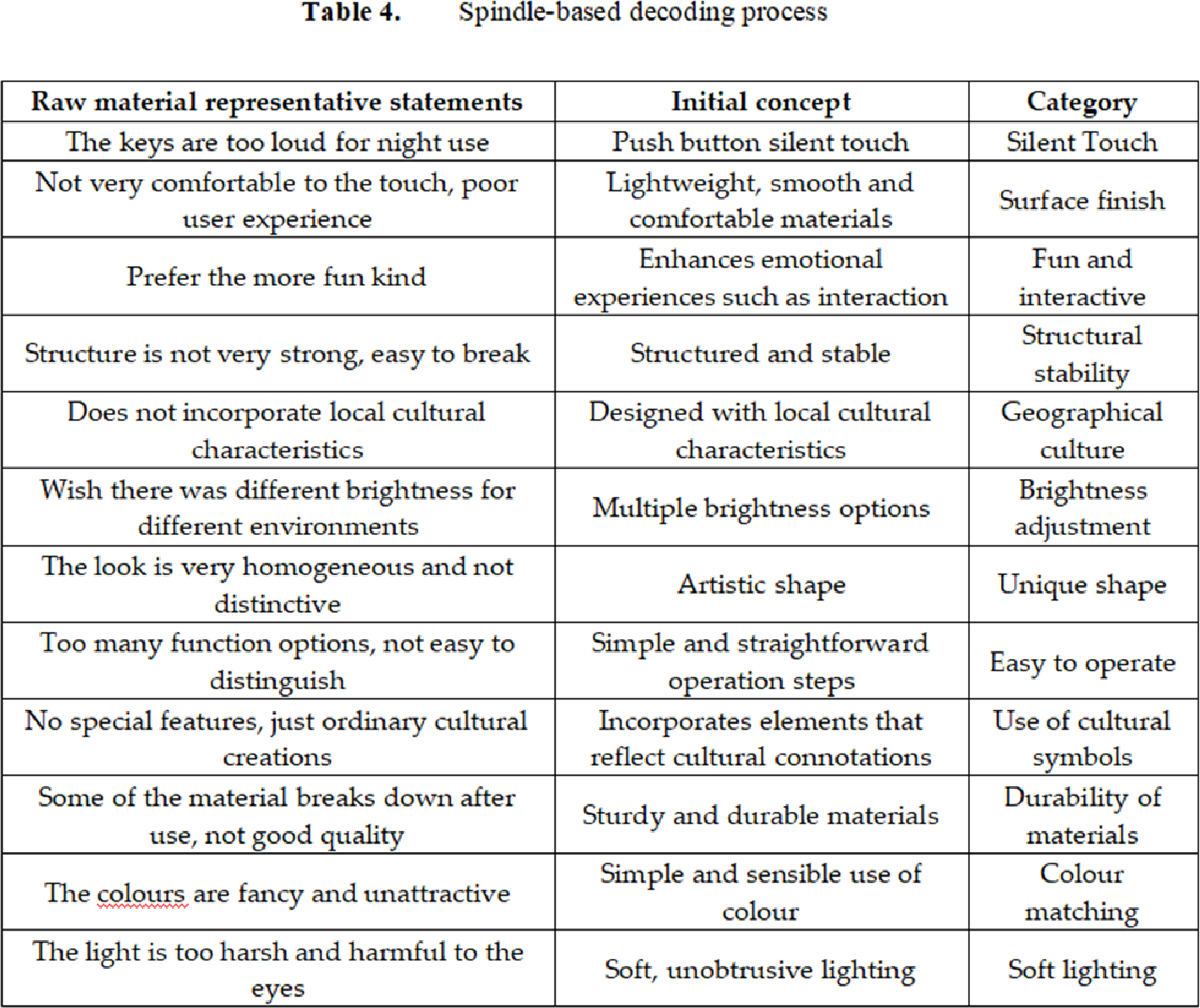


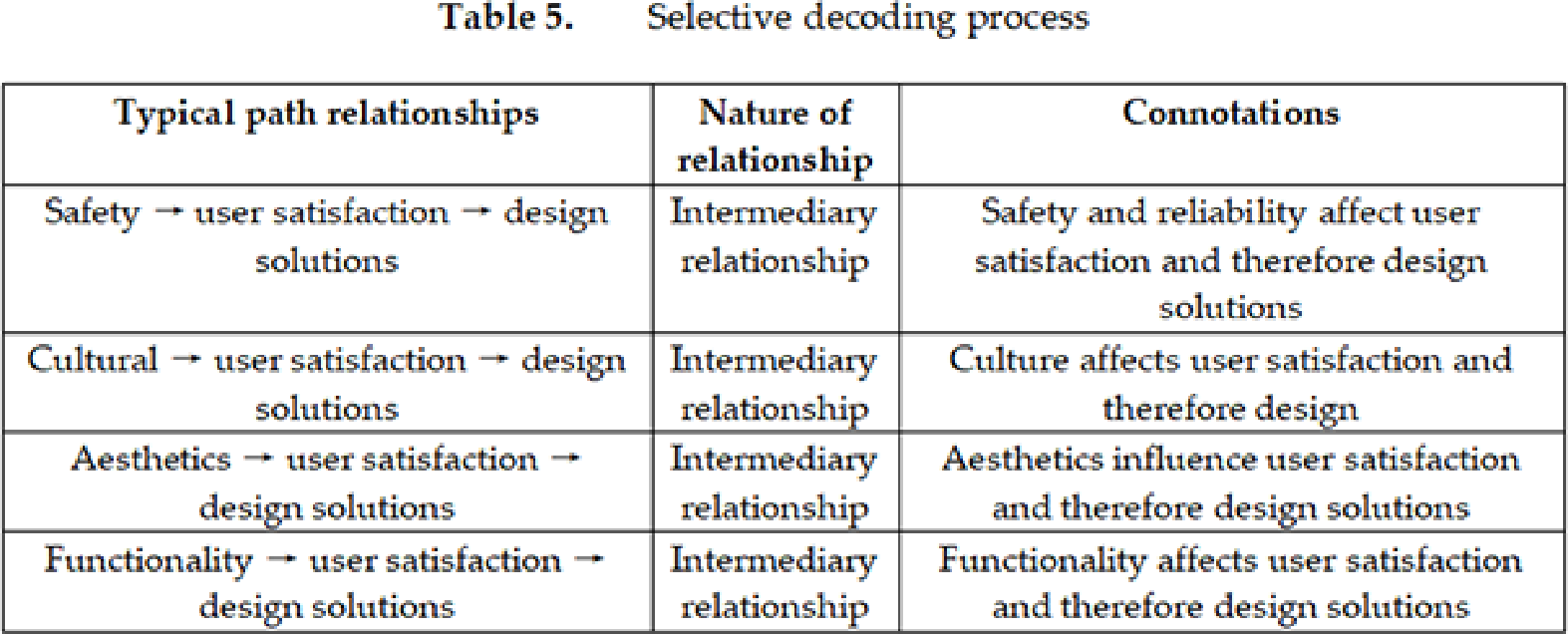


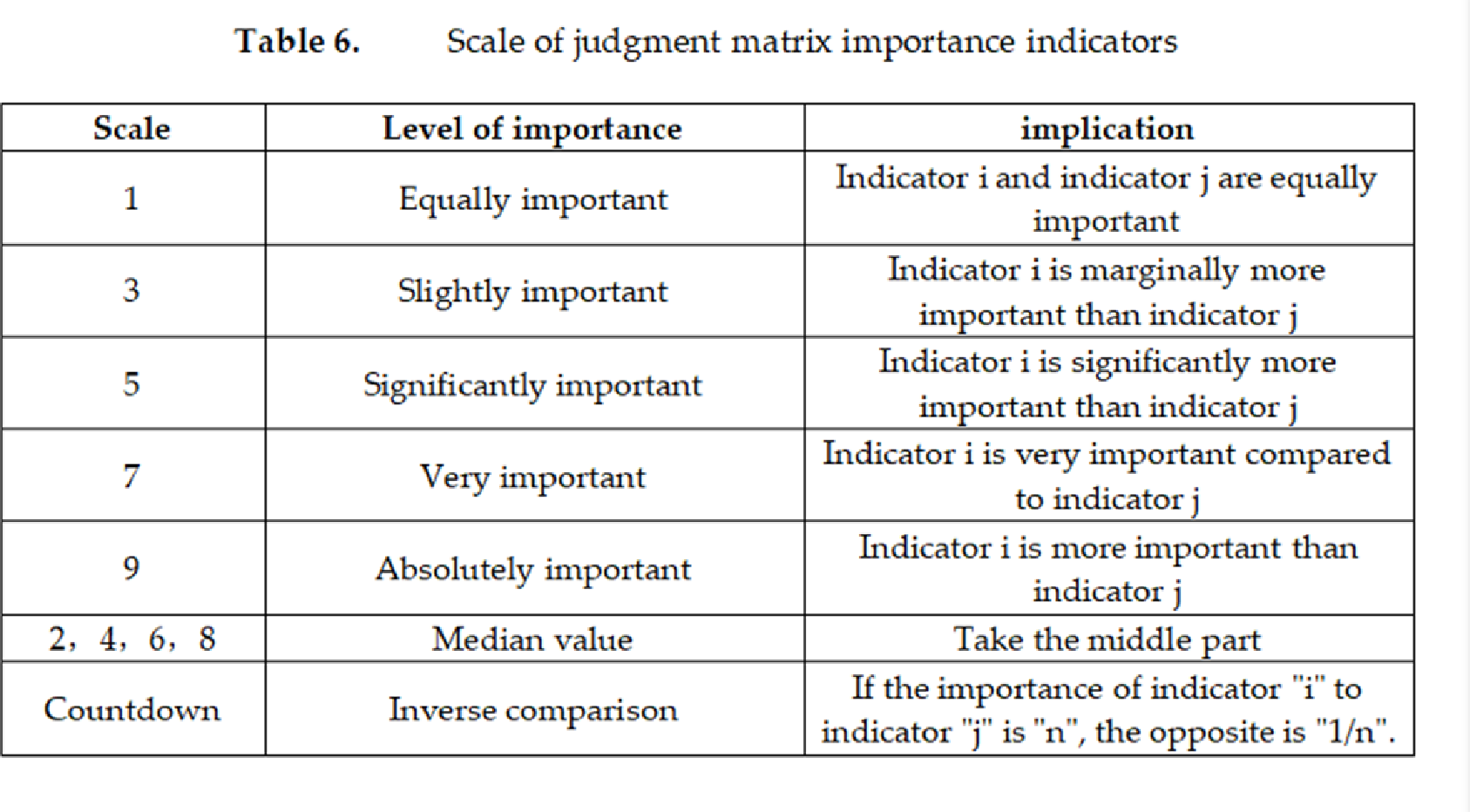


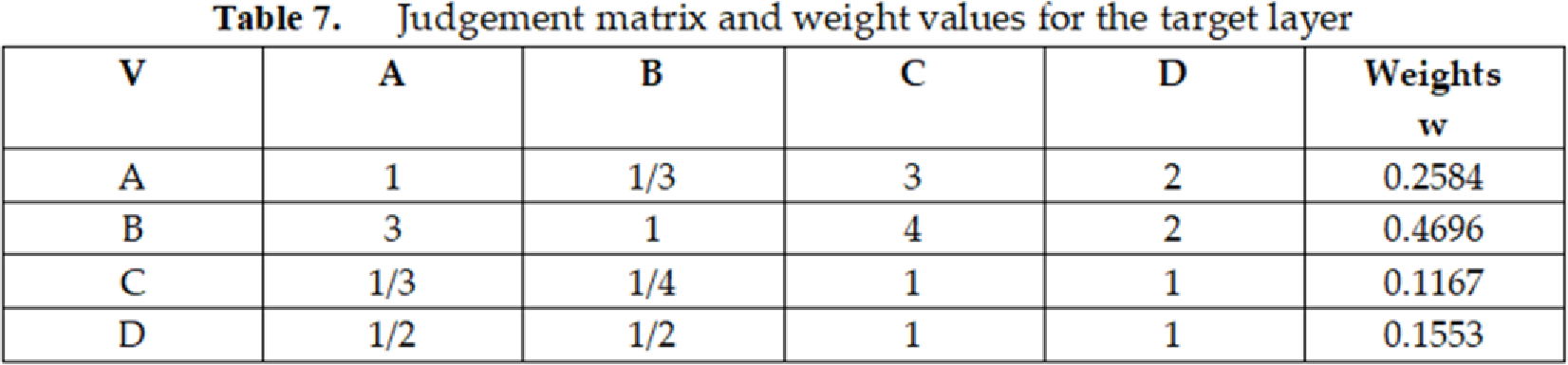


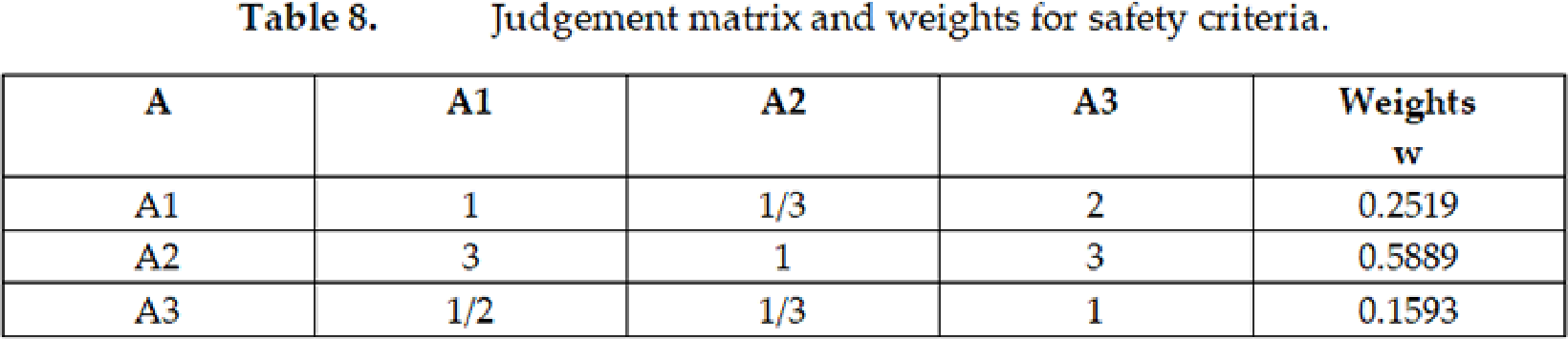


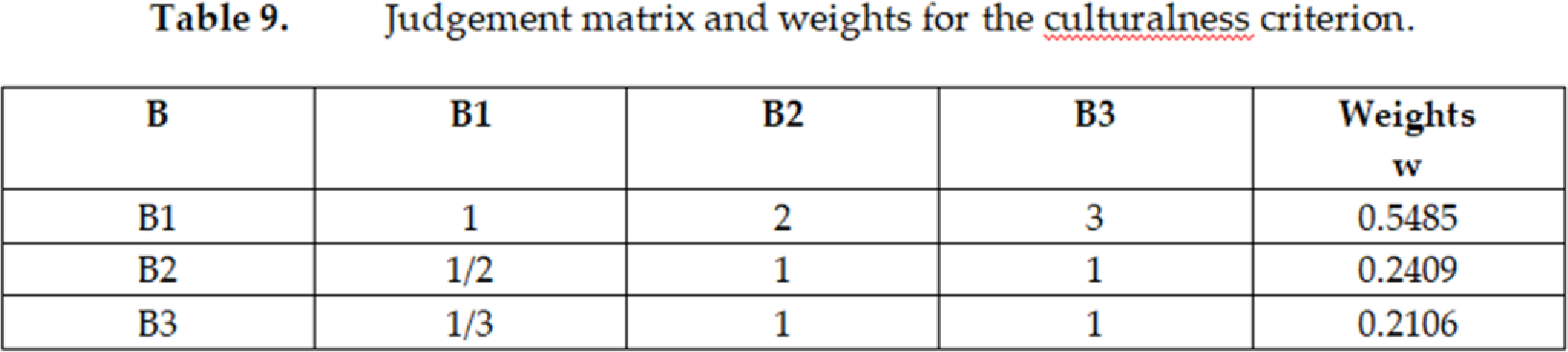


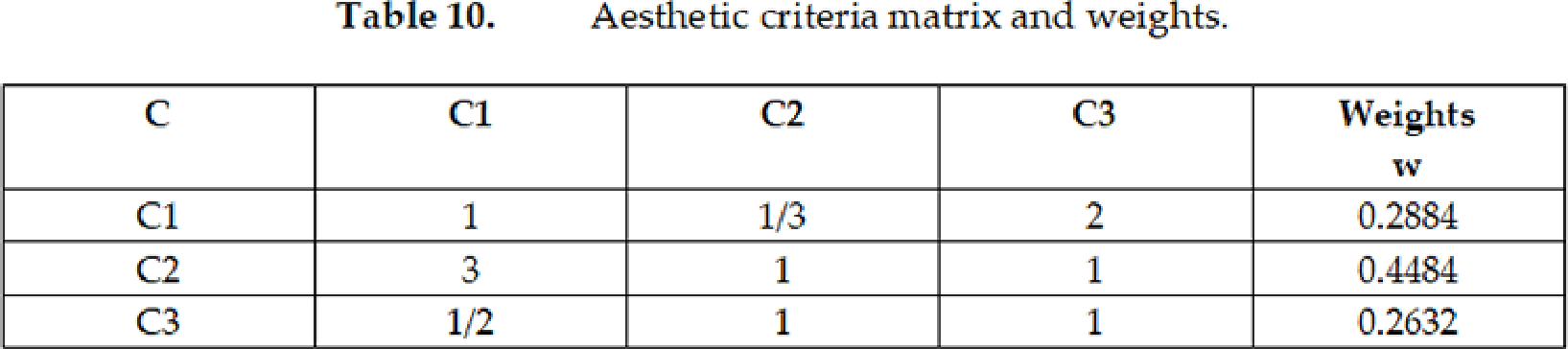


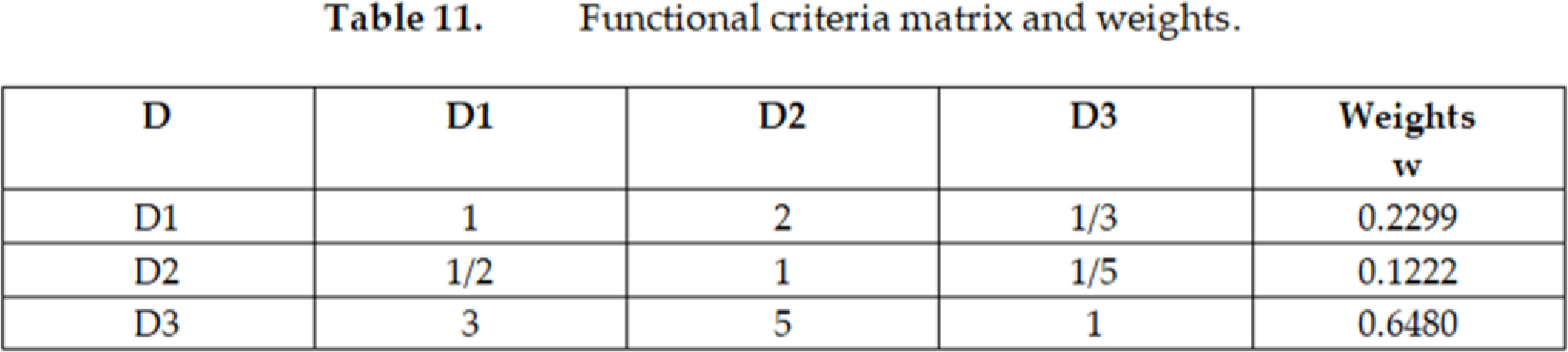


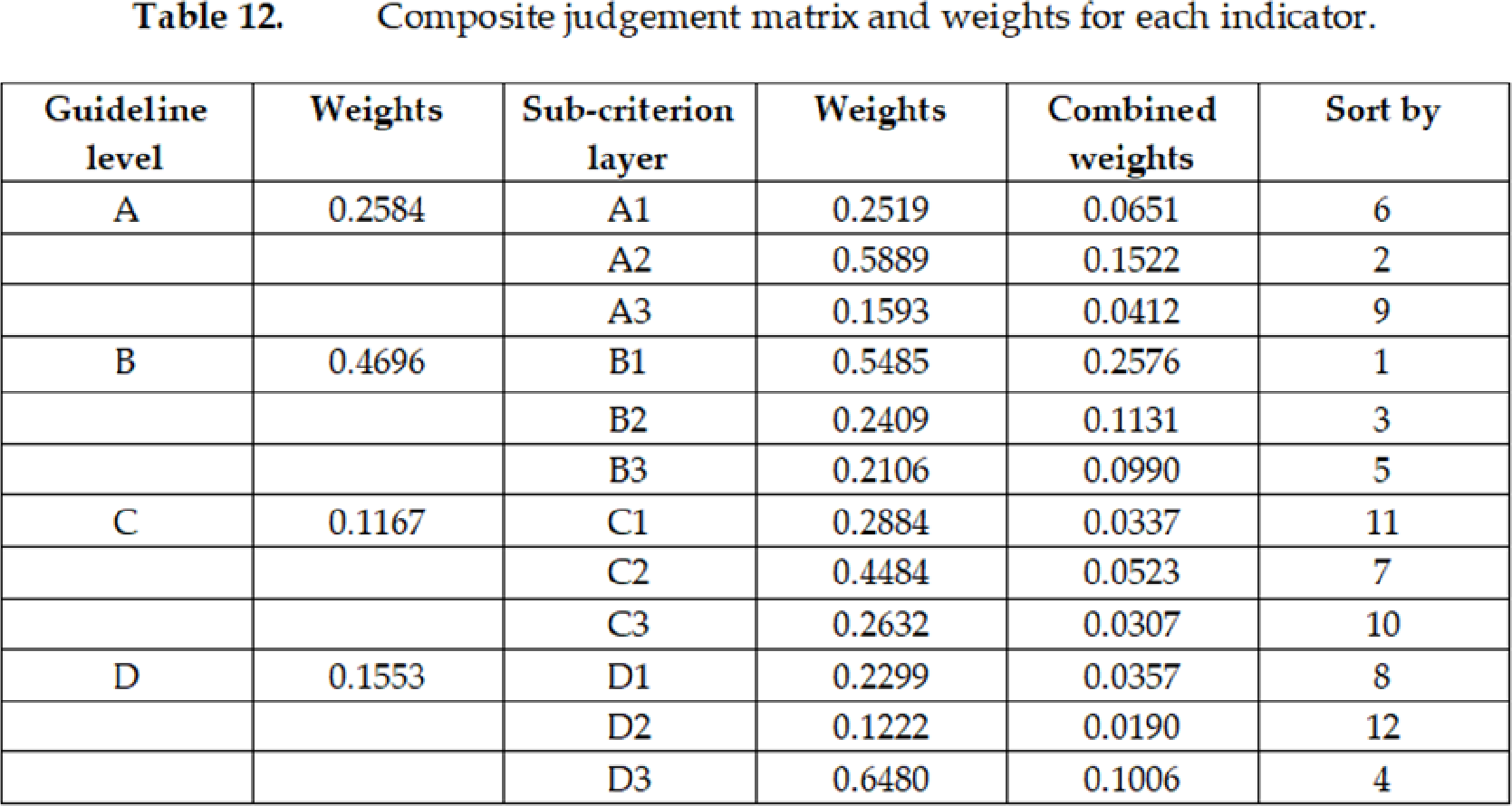


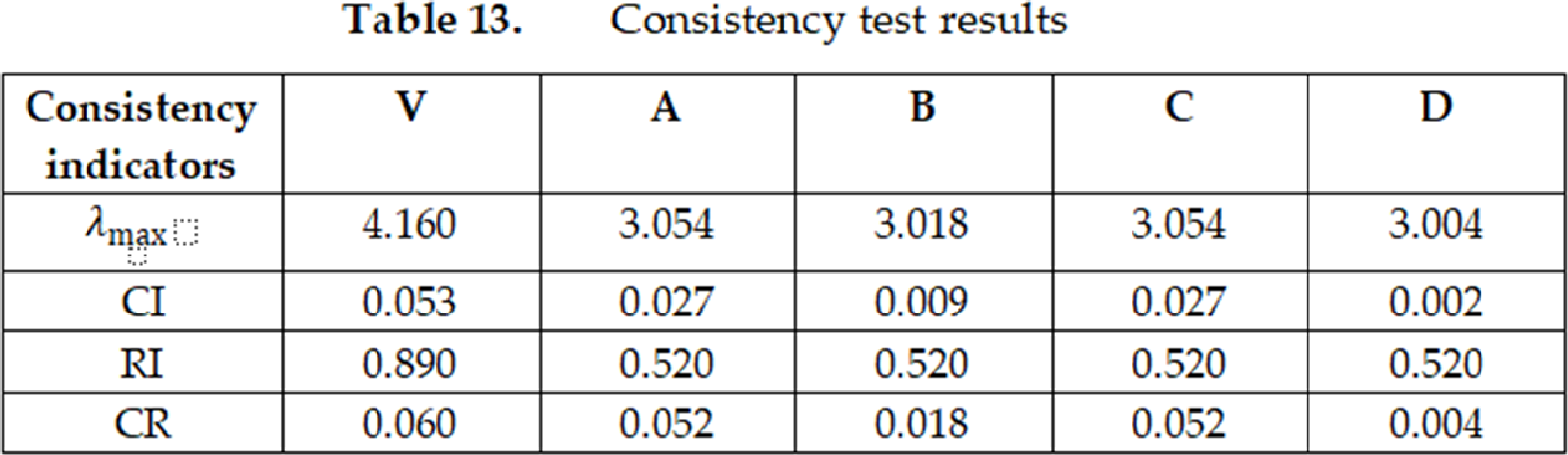


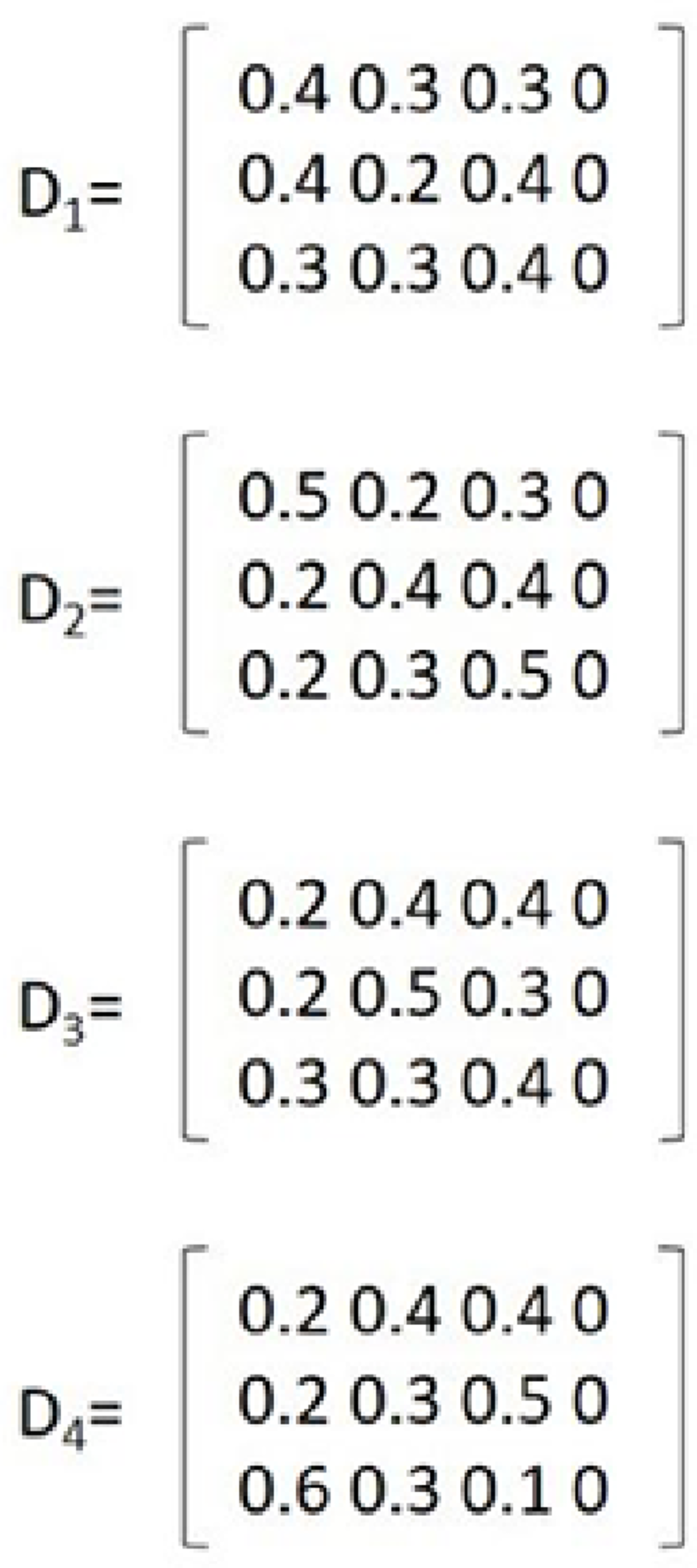


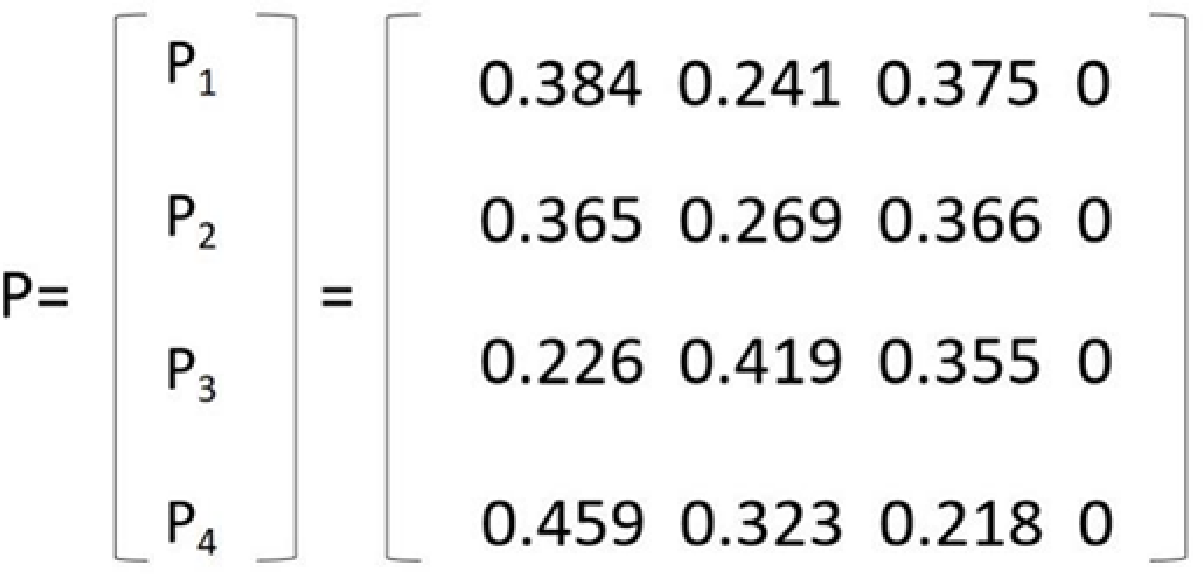

Supplement: S1 Table — (DOCX) [file pone.0286682.s004.docx]
